# Effects of Low‐Intensity Endurance Training on Aerobic Fitness and Risk Factors of Cardiometabolic Health in Working‐Age Adults: A Systematic Review and Meta‐Analysis

**DOI:** 10.1111/sms.70208

**Published:** 2026-01-16

**Authors:** Olli‐Pekka Nuuttila, Pekka Matomäki, Jani Raitanen, Harri Sievänen, Tommi Vasankari

**Affiliations:** ^1^ The UKK Institute for Health Promotion Research Tampere Finland; ^2^ Faculty of Sport and Health Sciences University of Jyväskylä Jyväskylä Finland; ^3^ Paavo Nurmi Centre & Unit for Health and Physical Activity University of Turku Turku Finland; ^4^ Faculty of Social Sciences (Health Sciences) Tampere University Tampere Finland; ^5^ Faculty of Medicine and Health Technology Tampere University Tampere Finland

**Keywords:** blood glucose, blood lipids, blood pressure, cardiorespiratory fitness, low‐intensity training, maximum oxygen uptake

## Abstract

There is a lack of meta‐analyses focusing on low‐intensity endurance training (LIT), including considerations of the lowest effective intensity across different outcomes. This systematic review and meta‐analysis examined the effects of LIT on aerobic fitness and cardiometabolic health. Randomized controlled trials involving healthy adults aged 18–65 were included if the training intervention was ≥ 3 weeks, intensity was exclusively below the first lactate/ventilatory threshold (VT1), or ≤ 60% heart rate reserve, or maximum oxygen uptake (VO_2max_), or ≤ 75% maximum heart rate. Outcome variables were VO_2max_, VT1, systolic and diastolic blood pressure, plasma/serum low‐density, high‐density, and total cholesterol, triglycerides, and glucose. Effect sizes (ES) were calculated according to Hedge's *g*. The subgroup analyses (*Q*‐test) examined the effects of training and background characteristics on outcomes. A total of 50 studies with 824 participants in the intervention groups were included. LIT had a large effect on relative VO_2max_ (ES = 0.94, *p* < 0.001, *I* ^2^ = 0.73) and a moderate effect (ES = 0.74, *p* = 0.003, *I* ^2^ = 0.57) on VT1 compared with the control group. Small but significant effects (|ES| = 0.29–0.44, *p* < 0.05, *I* ^2^ = 0.39–0.79) were observed for other variables, excluding glucose. According to the subgroup analysis, exercise intensity was associated with the adaptations only in VO_2max_ (*p* = 0.02). LIT improved aerobic fitness and cardiometabolic health, but effects on fitness were more pronounced. Although higher exercise intensity was associated with greater adaptations in VO_2max_, no minimum intensity for adaptations was detected for most outcomes. Notable heterogeneity in responses was observed, which likely reflects both methodological differences (e.g., intensity prescription) between studies and uncertainty regarding the response magnitude.

**Trial Registration:** The review protocol was registered at PROSPERO (CRD42023469528)

## 1 Introduction

Endurance training provides many positive effects not only on aerobic fitness but also on cardiometabolic health. The targeted positive effects are mostly modified by training dose, that is, the intensity, volume, and frequency of training [1]. General physical activity recommendations take into account both the volume and intensity of endurance activities, with separate weekly recommendations for moderate and vigorous intensities [2]. However, there are numerous ways to categorize exercise intensity, and these methods are different for general populations and athletic populations [3]. This fact has partly complicated the comparison of the actual effects of exercise intensity, as “moderate” intensity could practically mean 3–6 METs (metabolic equivalents) of absolute intensity [4], the exercise intensity domain below the first lactate or ventilatory thresholds (LT1 or VT1) [5], the exercise intensity domain between the first and the second LTs or VTs [5], or an arbitrary percentage, for example, 64%–76% of maximum heart rate (HR_max_) or 46%–63% of maximum oxygen uptake (VO_2max_) [3]. Previous meta‐analyses on moderate‐intensity training have also applied a wide range of actual exercise intensities, for example, < 65%/VO_2max_ [6], < 70%/VO_2max_ [7], and < 80%/VO_2max_ [8], while generally not emphasizing the LT1 or VT1 as a criterion for defining moderate intensity. Since acute physiological responses [9] and long‐term adaptations [10] can vary significantly across different intensity domains, it would be important to study the training effects specifically within a defined intensity range. To avoid terminological confusion, in this meta‐analysis, the term “low‐intensity training” (LIT) is used to describe training that is designated to occur below the LT1 or VT1.

The LIT domain is quite broad, and although the upper end of the domain can be clearly defined by LT1 or VT1, the lower end is ambiguous. In absolute terms, a level of 1.5 METs has been used to distinguish sedentary behavior from light‐intensity activities [4]. In relative terms, 37%/VO_2max_ and 57%/HR_max_ have been proposed as the lower limits of light‐intensity activities for the general population [3], and 50%/VO_2max_ and 60% HR_max_ as the lower limits of “LIT‐zone” for athletic populations [11]. Swain et al. [12] suggested that initial aerobic fitness level can affect the minimum intensity required for VO_2max_ adaptations, and in populations with lower fitness levels (i.e., VO_2max_ < 40 mL/kg/min), no definitive threshold was found. For serum lipids, slightly higher minimum intensity has been suggested (i.e., 75%/HR_max_) [13], meaning that the threshold for effective training might also depend on the viewpoint and targeted outcome. The upper range (i.e., zone 2) of the LIT domain has been suggested to be more effective than the lower range, although research evidence supporting this claim remains relatively limited [14], and a universal definition of such a zone is challenging [15]. Understanding better how training intensity within the whole LIT domain affects health and fitness‐related adaptations would be crucial for tailoring training recommendations more precisely and for encouraging sedentary populations, who would derive the most benefit from increased physical activity [16, 17].

The effectiveness of endurance training has often been studied through changes in VO_2max_, as it is considered an important determinant of endurance performance in athletic populations [18] and a strong predictor of morbidity, mortality and cardiovascular health in the general population [19, 20]. Several meta‐analyses have found that higher intensity is associated with greater adaptations in aerobic fitness [7, 21, 22], at least in the short term [23]. Regarding long‐term changes [23] and, for example, changes in cardiovascular risk profile, the intensity‐dependent effect is not as apparent [8, 24, 25]. Although high‐intensity training (HIT) has gained a lot of popularity, it can also be questioned whether high‐intensity activities will lead to long‐term adherence among untrained populations, as they are perceived as relatively demanding and unpleasant [26]. Since activities of daily living are performed mostly at the LIT domain [27], LIT would be a more feasible option compared with HIT, especially since people tend to avoid high intensities in free‐living conditions [28]. Given the distinct advantages of aerobic fitness [19] and the added risks posed by physical inactivity to cardiometabolic health [16], it would be important to identify accessible strategies to promote physical activity across the general population. Such efforts could also yield clinically significant benefits to public health.

The purpose of this systematic review and meta‐analysis of randomized controlled trials was to examine the effects of LIT interventions on aerobic fitness and risk factors of cardiometabolic health in working‐age healthy adults. Furthermore, the contribution of potential modifiers (training and background characteristics) was analyzed, and the intensity dependence of adaptations within the LIT domain was assessed.

## 2 Methods

This systematic review with meta‐analysis conformed to the PRISMA 2020 guidelines for methodology and reporting [29]. The review protocol was registered at PROSPERO (CRD42023469528).

### 2.1 Eligibility Criteria

This systematic review with meta‐analysis examined the effects of low‐intensity endurance training on healthy working‐age adults. Studies that were included for the analysis had to fulfill the following criteria:

1. The study measured at least one of the outcome variables (discussed in detail in Section 2.5).
2. The study was a randomized controlled trial that included at least one parallel intervention group and control group or cross‐over control and intervention periods.
3. Participants were adults aged 18–65 years.
4. Training intervention was at least 3 weeks in duration.
5. Training was performed according to a structured and predefined program.
6. Intensity of the training was exclusively below the LT1 or VT1, or if the training was not defined or reported in relation to these thresholds, intensity limits were ≤ 60%/VO_2max_, ≤ 60%/VO_2_ reserve, ≤ 75% HR_max_, or ≤ 60%/HR reserve (HRR). Various fixed boundaries for exercise intensity domains can be found across scientific literature [30], but the current thresholds were chosen as they were considered to be below the LT1 in most individuals according to an analysis from the authors' laboratory [31], and they also aligned with the ACSM criteria on the transition from moderate‐intensity to vigorous‐intensity physical activity [32].

Exclusion criteria for the studies were:

1. Unclear description of training program (intensity, frequency, and/or duration).
2. Training program included training modalities other than endurance training.
3. Outcome measurements were not assessed before and after the training intervention.
4. Participants with body mass index (BMI) higher than 35 kg/m^2^ were included in the intervention.
5. Participants had diagnosed diseases or disorders, or were recovering from a disease/condition, or received medication for cardiometabolic disease/condition.
6. Pregnant participants were included.
7. Participants were subject to changes in environmental conditions.
8. Inclusion of nutritional interventions, potential ergogenic devices/modalities, or pharmacological agents.

### 2.2 Data Sources and Search Strategy

A systematic literature search was performed in the databases of PubMed and SPORTDiscus. The search was limited to publication dates before October 31, 2023. The language was restricted to English, and only human participants were involved. In addition to the literature search, references cited in systematic review reports on the same or similar topic and studies that the authors already knew beforehand were analyzed. Phrases that were used in searches are provided in the Supporting Information.

### 2.3 Study Selection

Based on the inclusion and exclusion criteria, two independent reviewers (OPN and PM) performed two levels of article screening for their eligibility: (1) title and abstract, and (2) full texts. All disagreements were resolved by discussion.

### 2.4 Data Collection Process

**FIGURE 1:**
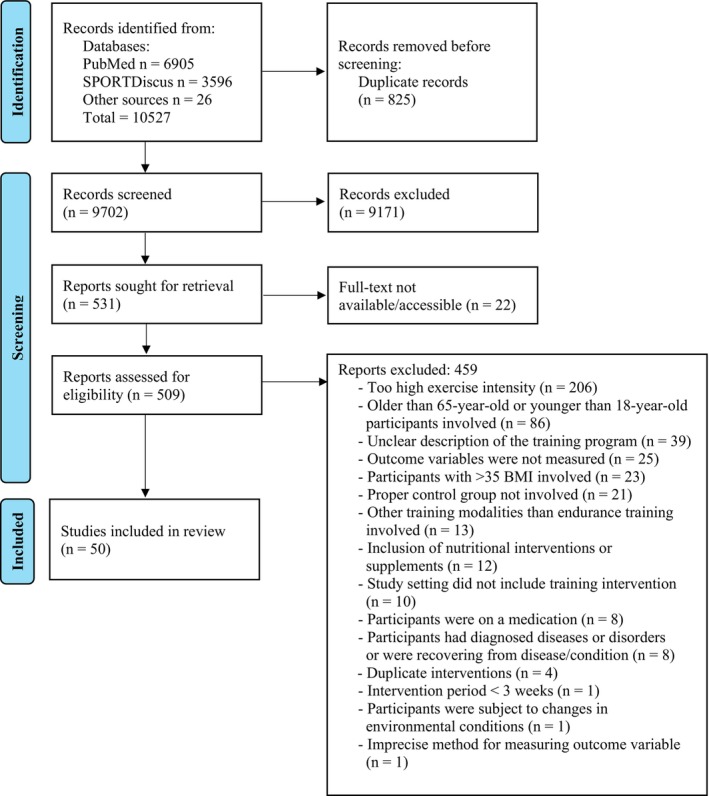
PRISMA flow diagram.

For each study fulfilling the inclusion criteria, two independent reviewers (OPN & PM) collected the data. All disagreements were resolved by a discussion. Extracted data comprised the first author's last name, year of publication, characteristics of participants (number, age, body mass, target population, BMI), characteristics of exercise intervention (intervention length, intensity, frequency, duration of training, training mode), methods for the testing of study outcomes (aerobic fitness, cardiometabolic health) and their mean results and standard deviation (SD) before and after the intervention. If data was available only in graphical form, WebPlotDigitizer software (version 4, Automeris LLC, Austin, USA) was used to extract the data points. If the necessary data was not reported in any form, the authors of the respective studies were contacted via email and/or ResearchGate to request the data. To minimize the risk of overlapping results from the same dataset, study designs, participant characteristics, and absolute results of each extracted study were compared. Four studies were excluded due to duplicate results (Figure 1).

### 2.5 Data Items

The collected variables included parameters that are associated with aerobic fitness and cardiometabolic health. Aerobic fitness parameters consisted of maximal (VO_2max_: absolute and relative to body mass, peak power in the cycle ergometer test [*P*_max_]) and submaximal (VO_2_ at the LT1 or VT1) variables. Cardiometabolic health parameters consisted of biomarkers that are related to cardiovascular and metabolic health. They were fasting total, high‐density lipoprotein (HDL) and low‐density lipoprotein (LDL) cholesterols, fasting plasma/serum glucose, fasting triglycerides, and systolic and diastolic blood pressure. From this point onward, all lipids and glucose are understood to be measured from fasting blood samples, either from serum or plasma. Study‐specific details can be found in Supporting Information. Of note, *P*_max_, total cholesterol, and LDL were not included in the original plan (CRD42023469528), but they were added as outcome variables. In turn, exercise economy was excluded from the analyses due to the lack of interventions measuring it (*n* = 1). Although some variables were not consistently measured or reported, all available outcomes were extracted from each included article. For the subgroup analyses, exercise intensity was transformed to %/VO_2max_ according to an equation proposed by Howley [33]: %/VO_2max_ = (%/HR_max_—29.95)/0.7305. %/HRR was considered equal to %/VO_2max_ as it was estimated to be a better approximation than %/VO_2_ reserve based on the results of Vehrs et al. [34].

### 2.6 Risk of Bias Assessment

Methodological quality and the risk of bias were assessed at a study level independently by two reviewers (OPN & PM) using the Cochrane Collaboration Risk of Bias 2.0 Tool [35]. The risk of bias was assessed in six domains: (1) bias arising from the randomization process, (2) bias due to deviations from intended interventions, (3) bias due to missing outcome data, (4) bias in measurement of the outcome, (5) bias in selection of the reported results, (6) other possible biases. Each of these categories was ranked for each study as having low, high, or unclear bias. Overall risk of bias was judged using the following criteria: Low risk of bias = The study was judged to be at low risk of bias for all domains for the given result. Some concerns = The study was judged to raise some concerns at least in one domain for the given result, but not to be at high risk of bias for any domain. High risk of bias = The study was judged to be at high risk of bias in at least one domain for the given result or the study was judged to have some concerns for multiple domains in a way that substantially lowers the confidence in the result.

### 2.7 Effect Measures

For the meta‐analysis, standardized mean differences according to Hedge's *g* formula and 95% confidence intervals (CIs) were calculated and presented as forest plots for all outcome variables. Further, 95% prediction intervals (PIs) were calculated for each outcome variable. The magnitude of Hedge's *g* (effect size = ES) was determined as per Cohen [36]: 0.2–0.49 = small, 0.5–0.79 = moderate, and at least 0.8 = large. ES < 0.2 was considered trivial. Equations used for ES analyses are provided in the Supporting Information.

### 2.8 Synthesis Method

Overall effects were analyzed with a random‐effects model with IBM SPSS Statistics v.29 (SPSS Inc., Chicago, IL, USA) using ES and variance of ES in each included study. The between‐study heterogeneity was analyzed with *I* ^2^ statistics, and 25%, 50%, and 75% were set as thresholds for low, medium, and high heterogeneity, respectively. Homogeneity was also analyzed with a *Q*‐test. Sensitivity analyses were performed with leave‐one‐out analysis. Minimum and maximum ES values after excluding single studies were analyzed for each outcome variable. Additionally, the effect of excluding the studies considered outliers (mean ± 2.68 × SD as suggested in [37]) or having a high risk of bias was assessed.

Subgroup analyses were conducted to analyze the effects of potential modifiers on outcome variables, including: (1) Baseline VO_2max_ (mL/kg/min: < 30, 30–40, > 40), (2) Training intensity (%/VO_2max_: < 50, 50–55, 55.1–60), (3) Duration of training sessions (0–30, 31–50, > 50 min), (4) Training volume (≤ 2.5, 2.6–4, > 4 h/week), (5) Frequency of training sessions (≤ 3/week, 3.1–4.9/week, ≥ 5/week), (6) Total endurance training dose (Banister's training impulse, TRIMP) [38] (arbitrary unit: ≤ 3.0, 3.1–4.4, ≥ 4.5), (7) Length of the training intervention (3–6, 7–11, ≥ 12 weeks), (8) Age (18–45, 45.1–65), (9) BMI (18.5–25, 25.1–35), (10) Sex (male, female). Rationale for each category is presented together with the results in Supporting Information. Subgroups were statistically compared with the between‐subgroup homogeneity *Q*‐test. Interventions were excluded from the subgroup analyses regarding the specified training characteristics if there was a progression in the training duration, frequency, or intensity from one subgroup to another. As an exception, “preparatory phases” of 1–2 weeks were allowed if there was no further progression afterwards. Interventions missing data for a specific subgroup variable (e.g., sex) were excluded from that subgroup analysis only, but they remained eligible for other analyses. In addition to the original research plan, a meta‐regression analysis was conducted to examine the effect of exercise intensity on the outcome variables (having ≥ 10 intervention groups), as the intensity dependence of adaptations was deemed one of the main questions of the current meta‐analysis. For these analyses, only studies with intensity progression of ≤ 5 percentage points (%/VO_2max_) were included. The Wald test was used as a model coefficient test in meta‐regression.

All statistical analyses were performed with SPSS v.29. The results of each variable are presented in order of magnitude.

### 2.9 Certainty of Evidence Assessment

The certainty of evidence was assessed with the Grading of Recommendations Assessment, Development, and Evaluation (GRADE) tool [39]. The risk of bias, inconsistency, indirectness of the evidence, imprecision, and publication bias (Egger's test) of each outcome variable was considered as not serious, serious, or very serious. GRADE was independently assessed by two authors (OPN and PM). All disagreements were resolved by discussion.

### 2.10 Descriptive Statistics

The absolute value and change of each variable were reported as the mean ± standard deviation.

## 3 Results

### 3.1 Study Selection

The study selection process is summarized in a PRISMA flow diagram (Figure 1). Out of the 9702 records screened, a total of 50 studies were included in the review.

### 3.2 Characteristics of the Included Studies

The characteristics of each included study [40, 41, 42, 43, 44, 45, 46, 47, 48, 49, 50, 51, 52, 53, 54, 55, 56, 57, 58, 59, 60, 61, 62, 63, 64, 65, 66, 67, 68, 69, 70, 71, 72, 73, 74, 75, 76, 77, 78, 79, 80, 81, 82, 83, 84, 85, 86, 87, 88, 89] are presented in Table S1. All studies consisted of parallel intervention (*n* = 54) and control (*n* = 50) groups. Exercise intensity was reported according to %/HR_max_ (66.5 ± 5.7) in 21 studies, according to %/VO_2max_ (53.2 ± 6.9) in 20 studies, and according to HRR% (54.0 ± 7.0) in eight studies. Only one study [61] used LT1 or VT1 for the determination of exercise intensity. Intensity was prescribed by the measured maximum value in 36 studies and by the estimated maximum value in 13 studies. Mean training frequency was 3.8 ± 1.1 sessions/week, and mean duration of sessions was 41 ± 13 min. Training interventions lasted on average 12.2 + 5.7 weeks. There was a progression in training frequency in eight studies. Training intensity progressed during the intervention in 10 studies and training session duration increased in 10 studies. The most frequently reported training modes were cycling (*n* = 19) and walking and/or running (*n* = 18). In addition, swimming (*n* = 1), and open choice from multiple modes (*n* = 11) were reported. Training mode was not clearly specified in one study [78].

In total, there were 824 participants in the intervention groups and 708 in the control groups. The number of male participants in the intervention groups was 441 and in the control groups 387. The respective numbers of female participants were 316 and 244. Four studies did not specify the number of males and females finishing the intervention. The mean age in the intervention groups was 35.5 ± 12.5 years, and in the control groups 33.2 ± 11.8 years. Participants were regarded mainly as sedentary or untrained, and none of the included studies reported results among trained/competitive athletes.

### 3.3 Risk of Bias in Studies

**FIGURE 2:**
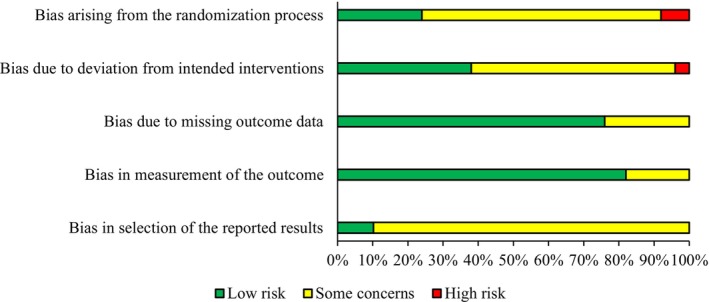
Risk of bias assessment results. Bars present domain‐specific proportion of studies having low risk (green), some concerns (yellow), and high risk (red).

The results of the risk of bias assessment are presented in Figure 2 and in Table S2. 86% of the studies had some concerns and 14% had a high risk of bias. Risks of bias were due to four main reasons: (1) The randomization process was not described in detail, and therefore it was not possible to assess whether it was performed appropriately (domain 1). (2) The adherence rate was not reported in 24 studies, and in three studies it could be considered low (< 80%), which could affect the outcomes (domain 2). (3) The study was not preregistered, and as a consequence, it was not possible to assess whether the results were analyzed according to a predefined plan (domain 5). (4) If exercise is considered a form of intervention, it is basically impossible to “blind” participants from the received intervention, which can be considered a risk of bias.

### 3.4 Meta‐Analysis

The mean values at baseline and LIT versus control group comparisons in the outcome variables are shown in Table 1. The absolute results of all individual studies are presented in Tables S3–S13, and the results of the subgroup analyses related to training and intervention characteristics are presented in Tables S14–S23.

**TABLE 1:** The mean ± SD absolute values for each outcome variables: Pre‐values, change and change between low‐intensity training (LIT) and control group. Mean and SD are calculated as weighted values.

Outcome variable	Number of intervention groups	Control pre	LIT pre	LIT vs. control (Δ)	ES
VO_2max_ (mL/kg/min)	37	32.5 ± 5.8	32.5 ± 5.5	4.5 ± 2.4	0.94*** (Large)
VO_2max_ (mL/min)	7	2610 ± 380	2800 ± 470	390 ± 190	0.84*** (Large)
P_max_ (W)	9	192 ± 33	193 ± 30	35 ± 11	1.09*** (Large)
VT1 VO_2_ (mL/kg/min)	4	19.2 ± 5.6	19.7 ± 4.8	2.3 ± 0.9	—
VT1 VO_2_ (mL/min)	1	1290 ± 360	1100 ± 150	390	—
VT1 pooled	5	—	—	—	0.74** (Moderate)
TC (mmol/L)	20	5.40 ± 0.83	5.51 ± 0.89	−0.19 ± 0.29	−0.29** (Small)
HDL (mmol/L)	21	1.26 ± 0.27	1.25 ± 0.30	0.09 ± 0.10	0.32*** (Small)
LDL (mmol/L)	15	3.75 ± 0.79	3.76 ± 0.89	−0.32 ± 0.31	−0.42*** (Small)
Gl (mmol/L)	12	5.24 ± 0.46	5.21 ± 0.39	−0.05 ± 0.29	−0.18 (Trivial)
TG (mmol/L)	19	1.38 ± 0.65	1.41 ± 0.62	−0.09 ± 0.18	−0.38*** (Small)
SBP (mmHg)	17	124.1 ± 12.2	122.9 ± 12.8	−4.0 ± 5.8	−0.41* (Small)
DBP (mmHg)	17	78.9 ± 8.9	78.3 ± 9.0	−3.6 ± 4.0	−0.43*** (Small)

Abbreviations: DBP, diastolic blood pressure; Gl, glucose; HDL, high‐density lipoprotein cholesterol; LDL, low‐density lipoprotein cholesterol; SBP, systolic blood pressure; TC, total cholesterol; TG, triglycerides; VO_2_, oxygen uptake; VO_2max_, maximum oxygen uptake; VT1, the first ventilatory threshold; P_max_, peak power in the cycle ergometer test.

* *p* < 0.05.

** *p* < 0.01.

*** *p* < 0.001.

#### 3.4.1 Effect of LIT on Aerobic Fitness

**FIGURE 3:**
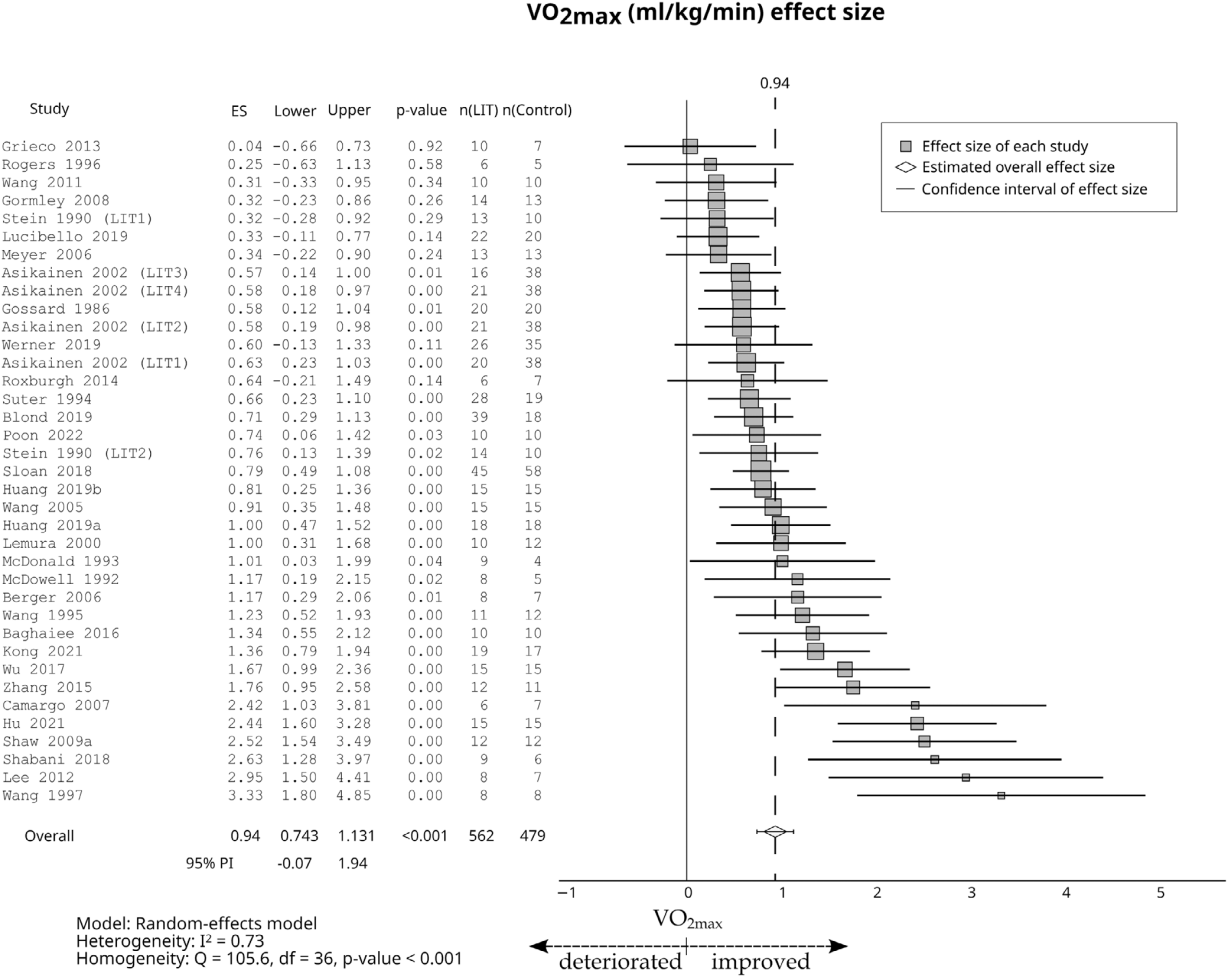
The effects of LIT on VO_2max_ (mL/kg/min) compared with the control group. PI, prediction interval; VO_2max_, maximum oxygen uptake.

**FIGURE 4:**
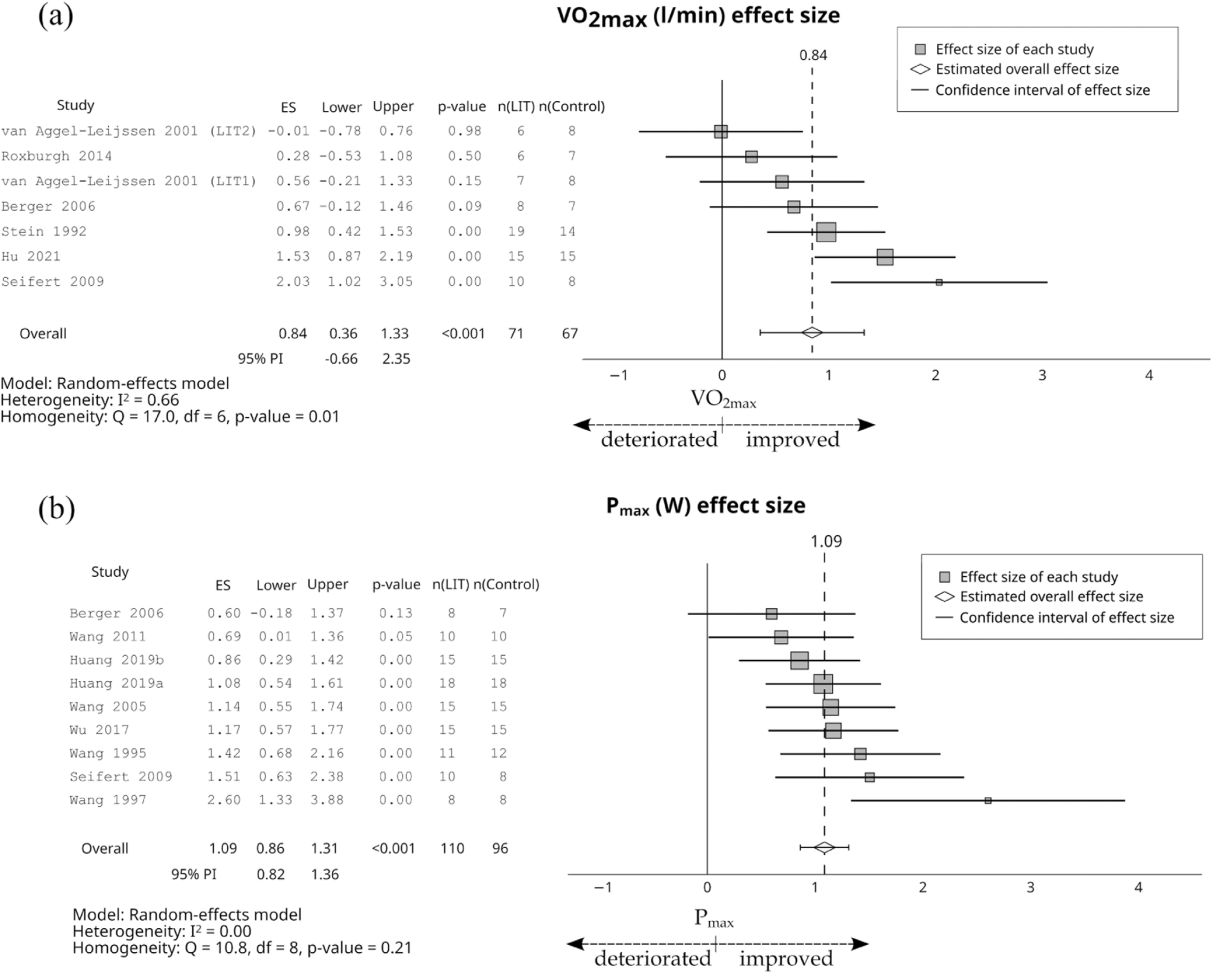
The effects of LIT on (a) VO_2max_ (L/min) and (b) *P*_max_ compared with control group. PI, prediction interval; *P*_max_, peak power in the cycle ergometer test; VO_2max_, maximum oxygen uptake.

**FIGURE 5:**
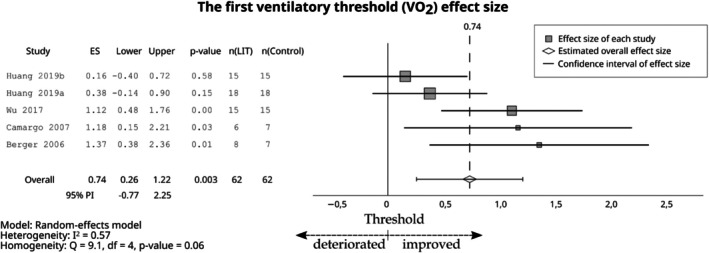
The effects of LIT on VT1 compared with the control group. PI, prediction interval; VT1, The first ventilatory threshold.

Compared with the control group, LIT had a large effect on relative VO_2max_ (ES = 0.94; 95% CI 0.74–1.13; 95% PI −0.07 to 1.94, Figure 3), absolute VO_2max_ (ES = 0.84; 95% CI 0.36–1.33; 95% PI −0.66 to 2.35, Figure 4), and *P*_max_ (ES = 1.09; 95% CI 0.86–1.31; 95% PI 0.82–1.36, Figure 4). The effect of LIT was moderate on the VT1 (ES = 0.74; 95% CI 0.26–1.22; 95% PI −0.77 to 2.25, Figure 5).

#### 3.4.2 Effect of LIT on Cardiometabolic Variables

**FIGURE 6:**
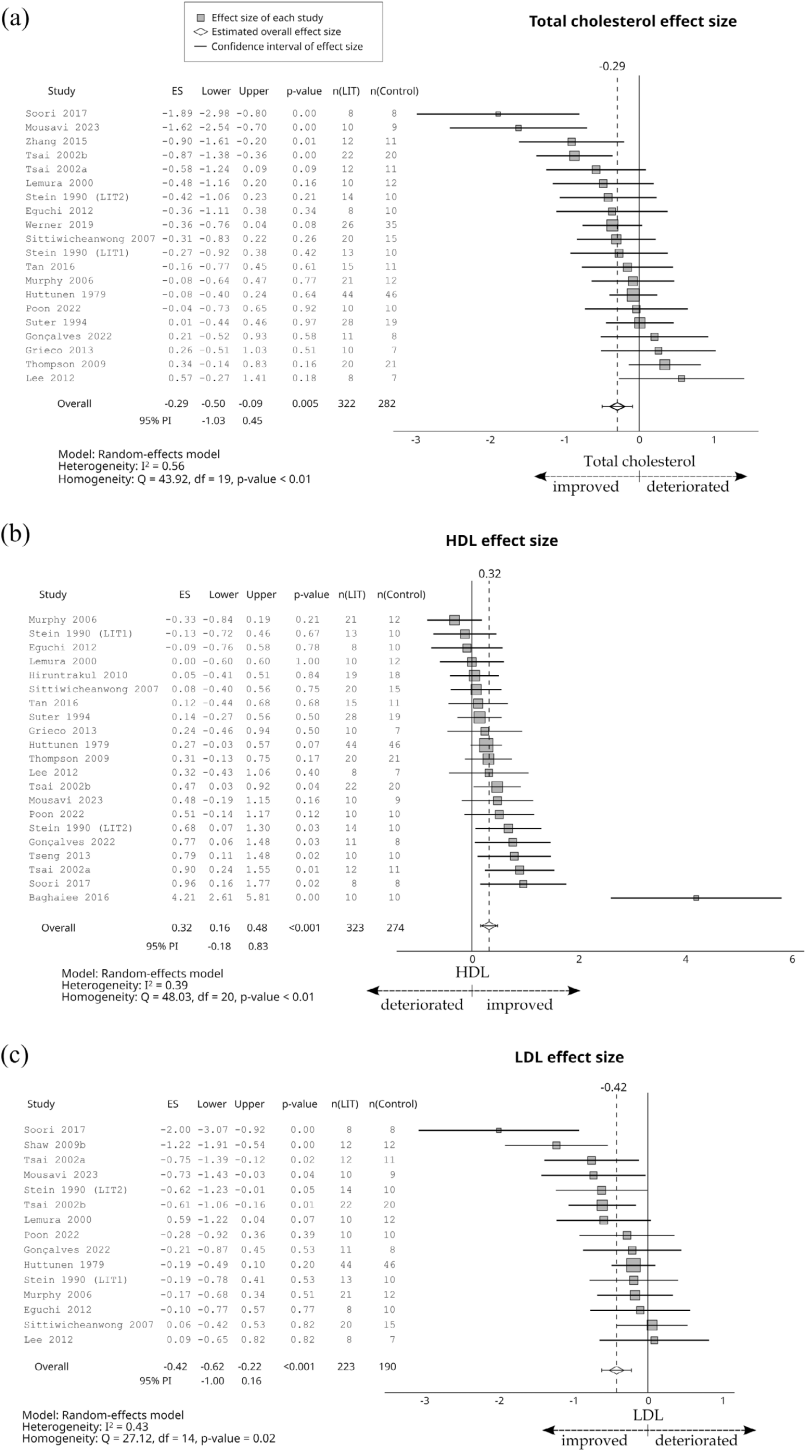
The effects of LIT on (a) total cholesterol, (b) HDL, and (c) LDL compared with the control group. PI, prediction interval.

**FIGURE 7:**
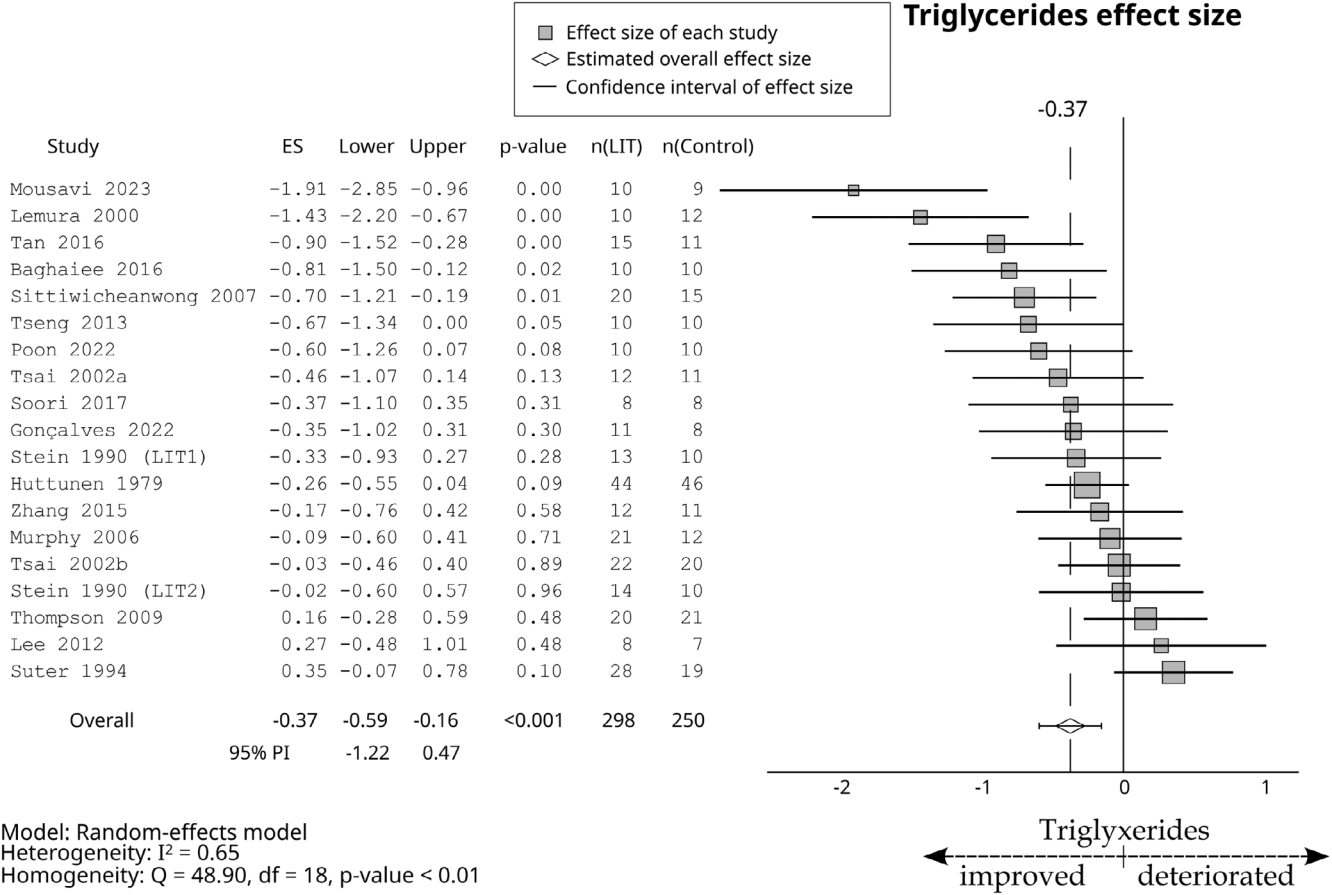
The effects of LIT on triglycerides compared with the control group. PI, prediction interval.

**FIGURE 8:**
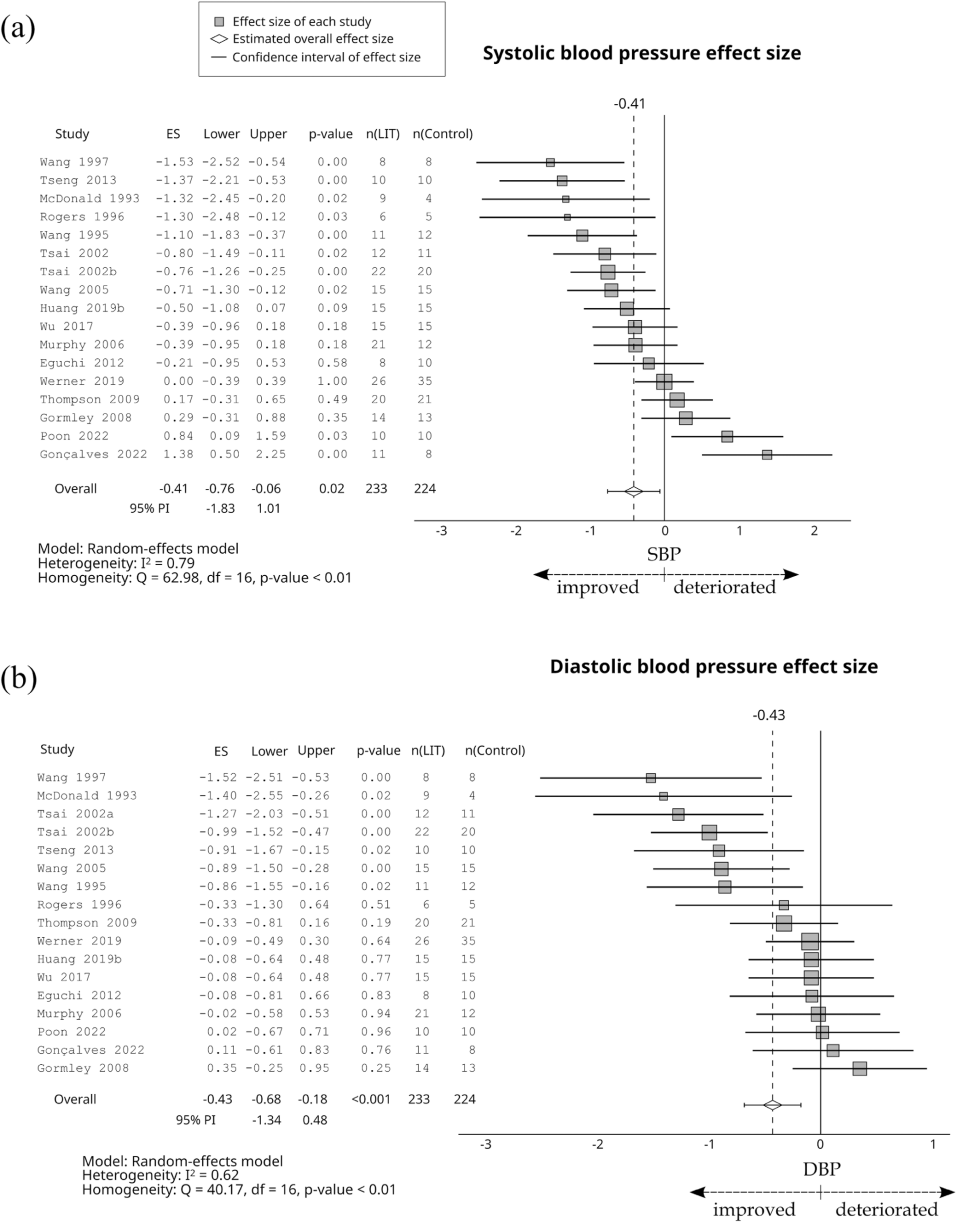
The effects of LIT on systolic and diastolic blood pressures compared with the control group. PI, prediction interval.

**FIGURE 9:**
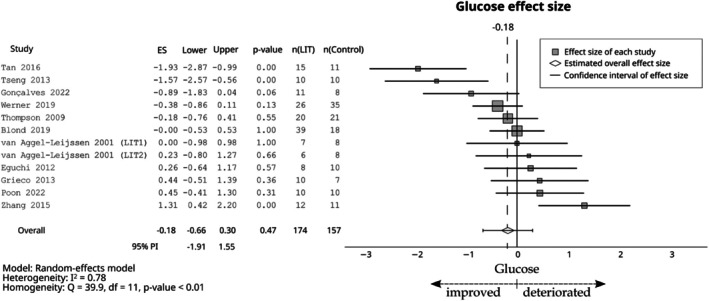
The effects of LIT on glucose compared with control group. PI, prediction interval.

Compared with the control group, LIT had a small negative effect on total cholesterol (ES = −0.29; 95% CI −0.50 to −0.09; 95% PI −1.03 to 0.45, Figure 6), LDL (ES = −0.42; 95% CI −0.62 to −0.22; 95% PI −1.00 to 0.16, Figure 6), triglycerides (ES = −0.38; 95% CI −0.60 to −0.16; 95% PI −1.22 to 0.47, Figure 7), systolic blood pressure (ES = −0.41; 95% CI −0.76 to −0.06; 95% PI −1.83 to 1.01, Figure 8), and diastolic blood pressure (ES = −0.43; 95% CI −0.68 to −0.18; 95% PI −1.34 to 0.48, Figure 8). Furthermore, a small positive effect was observed on HDL (ES = 0.32; 95% CI 0.16–0.48; 95% PI −0.18 to 0.83, Figure 6). The effect was trivial for glucose (ES = −0.18; 95% CI −0.66 to 0.30; 95% PI −1.91 to 1.55, Figure 9).

#### 3.4.3 Effects of Training Characteristics on Adaptations

**FIGURE 10:**
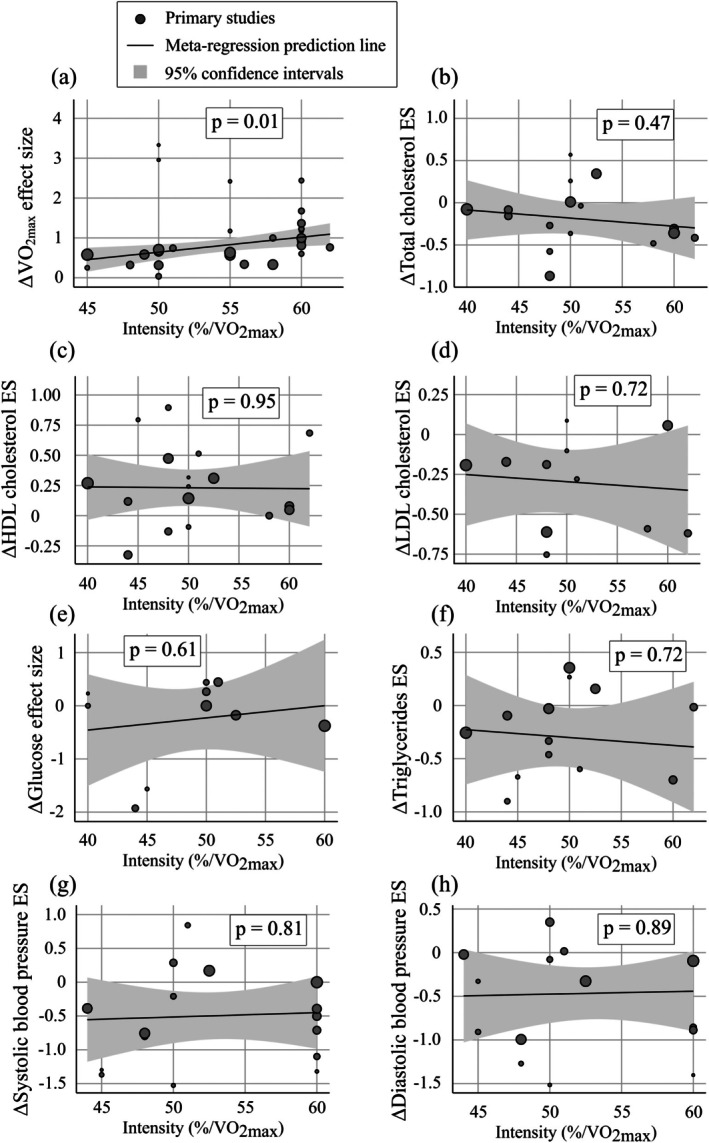
Meta‐regression figures with intensity as covariate for the change in (a) VO_2max_, (b) total cholesterol, (c) HDL, (d) LDL, (e) glucose, (f) triglycerides, (g) systolic blood pressure, and (h) diastolic blood pressure. *p*‐value represents the value of the model coefficient test.

Several significant differences (*p* < 0.05) were found in the subgroup analyses, but the dose–response associations between training characteristics and training adaptations were effective only regarding relative (*p* = 0.021) and absolute (*p* = 0.03) VO_2max_ responses to intensity (Tables S14–S23). This was further supported by the meta‐regression (Figure 10), which revealed that within the examined low‐intensity domain, only relative VO_2max_ had an intensity‐dependent response (*p* = 0.01).

#### 3.4.4 Effects of Participant Characteristics on Adaptations

Baseline VO_2max_ had a significant effect (*p* < 0.001) on the systolic blood pressure response (< 30 mL/kg/min: ES = −1.43, 95% CI −2.19 to −0.67; 30–40 mL/kg/min: ES = −0.17, 95% CI −0.56 to 0.22, Table S22). In turn, age had an effect (*p* < 0.008) on VO_2max_ response (≤ 45 years: ES = 1.04, 95% CI 0.79–1.30; 45.1–65 years: ES = 0.63, 95% CI 0.45–0.80, Table S14). Based on subgroup analyses, sex or baseline BMI did not have a significant effect on any of the assessed outcomes.

#### 3.4.5 Results of Sensitivity Analyses

Sensitivity analyses were performed by repeating the ES analyses while excluding the studies that were considered as outliers or whose methodological characteristics could have potentially influenced the results. Two results fulfilled the predefined 2.68 × SD criterion (one in HDL and one in LDL). Exclusion of these results did not affect HDL (ES = 0.27, 95% CI 0.14–0.40; 95% PI 0.01–0.54) or LDL (ES = −0.36, 95% CI −0.54 to −0.19; 95% PI −0.78 to 0.05) results significantly. When the studies with high risk of bias (*n* = 7), or studies where the intensity was prescribed by the estimated maximum value (*n* = 13) were excluded, no differences were found in the magnitude or statistical significance of any of the outcomes (data not shown). Leave‐one‐out analysis indicated no changes in statistical significances or magnitude of ES (Tables S24 and S25).

### 3.5 Certainty of Evidence

The certainty of evidence was analyzed according to GRADE (Table S26). It was considered high (grade 4/4) for VO_2max_ (absolute and relative) and *P*_max_, moderate (grade 3/4) for VT1, total cholesterol, LDL, and diastolic blood pressure, and low (grade 2/4) for HDL, glucose, triglycerides, and systolic blood pressure. Egger's test suggested potential publication bias in HDL (*p* = 0.01) and triglycerides (*p* = 0.04) (Table S26).

## 4 Discussion

This systematic review and meta‐analysis showed that low‐intensity endurance training had moderate to large mean effects on maximal (VO_2max_, *P*_max_) and submaximal (VT1) aerobic fitness compared with controls. Simultaneously, small but statistically significant effects were found for the cardiometabolic variables (total cholesterol, HDL, LDL, triglycerides, and blood pressure). The subgroup analyses indicated that a higher exercise intensity was associated with greater improvements in VO_2max_, but no minimum effective intensities were found for assessed outcomes. Current results suggest that aerobic fitness and the cardiometabolic health profile can be significantly improved with relatively moderate training doses in terms of intensity and volume. However, the high heterogeneity in response magnitude across studies should be acknowledged.

### 4.1 Effects of LIT on Aerobic Fitness

It is well known that aerobic fitness (e.g., VO_2max_) can be improved with a wide range of exercise intensities [90]. Previous meta‐analyses on the effects of exercise intensity have suggested that HIT is more beneficial than LIT for improving aerobic fitness [7, 21, 22]. On the other hand, recent meta‐analysis of Mølmen et al. [23] did not find significant intensity‐dependent responses for VO_2max_ in longer interventions (i.e., > 10 weeks). The current meta‐analysis found systematic and large increases in VO_2max_ and *P*_max_, while for VT1 the effect was moderate. The mean magnitude of change in VO_2max_ (3.9 mL/kg/min) in LIT interventions was so substantial that it would in practice diminish the health risks of individuals with poor fitness [19]. For example, one MET (corresponding to 3.5 mL/kg/min oxygen uptake) increase in VO_2max_ has been suggested to decrease the risk for all‐cause mortality by 11%–17% and for heart failure by 18% [20]. Similar associations have also been reported for major adverse cardiac events among 40–60‐year‐old adults without a history of cardiovascular disease [91], highlighting the clinical relevance of improved aerobic fitness by itself. Although the pooled effects for fitness‐related outcomes were moderate‐to‐large in magnitude, at the same time, the prediction intervals remained relatively wide. This highlights the heterogeneity of the results, and it means that in practice not all studies (nor individuals) would achieve positive responses. The prediction intervals were the narrowest for the *P*_max_, indicating that maximum aerobic performance is the outcome most likely yielding (large) positive effect in future studies.

Swain and Franklin [12] have earlier proposed that 50%/VO_2max_ is the minimum effective intensity improving aerobic fitness for individuals with VO_2max_ above 40 mL/kg/min, while no such threshold exists for those below this level. The current findings support this, as all included exercise intensities induced positive effects compared with control groups. The lowest intensity included in the current meta‐regression analyses was 45%/VO_2max_, which aligns with the light‐to‐moderate‐intensity transition used by ACSM [32]. While no actual minimum intensity for VO_2max_ improvements was found, it cannot be concluded whether even lower exercise intensities could be effective. Unfortunately, the current study could not examine the effect of initial VO_2max_ across a broad range, as only a very limited number of studies (*n* = 3) included groups with a mean greater than 40 mL/kg/min.

An important notion in the subgroup analysis was that comparable positive effects were found across < 6‐week and > 12‐week interventions. This most likely highlights the rapid occurrence of many adaptations in sedentary or untrained individuals, but also the need for progression in training dose (intensity and/or volume), if further adaptations are desired. Contrary to the current findings, the meta‐analysis of Mølmen et al. [23] found a distinct effect of intervention duration on VO_2max_. The explanation for different results might be that their continuous training group also included studies using intensities well above the LT1 or VT1. It is possible that the inclusion of higher exercise intensities is necessary for long‐term improvements in aerobic fitness.

### 4.2 Effects of LIT on Risk Factors of Cardiometabolic Health

Endurance training has been shown to have positive effects on lipid profile and blood pressure [25, 92]. The link between aerobic fitness and the levels of plasma lipids [93] as well as the general risk of cardiovascular diseases is also broadly acknowledged [19]. The current results supported the exercise‐induced positive effects, although the magnitude of response was smaller in cardiometabolic variables compared with fitness‐related variables, and based on prediction intervals, there is more uncertainty in the magnitude of effect. While the changes in blood pressure were consistent with previous meta‐analysis [92], the findings regarding serum lipids did not fully align with earlier assumptions. Specifically, the present results challenge the claim by Fikenzer et al. [13] that “effective endurance training” at intensities of at least 75%–85% HR_max_ is required to induce beneficial changes in serum lipid profiles. This difference could relate to diverse background and training characteristics in the groups of “non‐effective training” in their analyses.

Regarding lipids, an increase in HDL has been a typical observation after endurance training interventions [25, 94]. In the current analysis, the greatest changes were found in the concentration of LDL (−9% = −0.32 mmol/L) and HDL (+7% = +0.09 mmol/L), while the change of TC was only −3% (= −0.19 mmol/L). From the clinical point of view, the change in LDL and HDL can also be considered more significant than the decrease of TC, as the magnitude was sufficient to influence the cardiovascular risk factors. For example, it has been suggested that every 0.026‐mmol/L increase in HDL reduces coronary heart disease risk by 2%–3% and cardiovascular disease mortality risk by 3.7%–4.7% [95], while for every 0.26 mmol/L reduction in LDL the relative risk reduction is 7.1% for coronary heart disease events and 4.4% for total deaths [96]. The benefits of LDL lowering seem apparent also among low‐risk vascular disease population [97]. The increase of the concentration of HDL is important, because pharmacological options to increase the level of HDL are limited. The magnitude of change in blood pressure observed in the current study could also be regarded as clinically relevant. This observation is further supported by Canoy et al. [98] who reported in their recent review a significantly reduced risk of cardiovascular events (10%) for every 5‐mmHg decrease in systolic blood pressure. Most importantly, the association was apparent even in normotensive individuals.

When interpreting the results, it is relevant to acknowledge that the current participants can be assumed to be generally healthy. For example, none of the studies reported mean results above the reference range of blood glucose as defined by WHO [99]. Based on previous studies, the responses would probably have been different and greater in magnitude among individuals at a higher cardiometabolic risk at baseline [25]. This is likely to affect prediction intervals as well because they indicated a lower certainty of positive changes in cardiometabolic variables compared with those in aerobic fitness. Regarding the clinical relevance of aforementioned changes in risk factors of cardiometabolic health, it should be noted that there is also a time‐related cumulative effect of these factors [100, 101]. Thus, even if the levels are only maintained without further adaptations, positive changes can still reduce the long‐term risk of cardiometabolic diseases. In addition, the total clinical benefit for cardiometabolic health could be greater than small when small beneficial effects take place simultaneously at several cardiometabolic risk factors (e.g., blood pressure, HDL, and TC) [102].

Another important consideration in interpreting the results is how to distinguish the effects of increased amount of physical activity, body mass change, and fitness change, as each could independently impact cardiometabolic health [103]. Furthermore, recent meta‐analysis found that each additional 30 min/week of aerobic exercise was associated with body mass reduction of 0.5 kg [104], demonstrating the potential inter‐reliance between these changes in training interventions. Although changes in body mass were also reported in many studies of the present meta‐analysis, these changes were generally considered trivial in magnitude and thus unlikely to have a significant impact on the results. In addition, based on the subgroup analyses, changes in any of the outcome variables were not different between overweight/obese (BMI from 25 to 35 kg/m^2^) and normal‐weight individuals (BMI < 25 kg/m^2^). Although the root cause of the observed cardiometabolic changes cannot be definitively determined, it is likely that they were moderated by all these factors.

### 4.3 Contribution of Exercise Frequency, Intensity and Duration

In general, it was somewhat unexpected how little impact the training and intervention characteristics had on the assessed outcomes. This observation contrasts with the findings of a recent meta‐analysis on endurance training [23]. The exercise intensity within the LIT domain seemed the most relevant characteristic, as higher intensity was associated with greater improvement in VO_2max_. This result lends support to the assumption that specifically the upper range of the LIT domain (i.e., zone 2) may promote fitness‐related adaptations [14]. Similar findings were reported in the meta‐analysis of Huang et al. [105] who found the most linear dose–response association with the intensity and VO_2max_ improvement (up to 75%/HRR) in older adults. However, the association was not similarly linear regarding exercise duration, frequency, or intervention duration. Notably, intensity or other training characteristics were not systematically associated with the changes in health‐related variables; thus, in sedentary and untrained populations, additional physical activity and exercise are likely to have positive effects quite rapidly in these outcomes regardless of the type of training performed. In turn, longer intervention durations do not necessarily induce greater effects, at least without progression or variation in training. In addition to being observed for LIT in the present meta‐analysis, this phenomenon has previously been noted for aerobic fitness after high [106], and very intensive supramaximal [107] training, where the adaptations plateaued after 2–10 weeks [23].

Some of the current subgroup results seemed contradictory, suggesting the greatest improvements with the shortest exercise durations and the smallest training volumes (e.g., total cholesterol), which could at least partly relate to the large heterogeneity in study characteristics. Furthermore, the small sample size of some subgroups should be acknowledged, as it decreased the statistical power of the *Q*‐test in the moderator analysis. Therefore, to avoid incorrect conclusions, these results should be interpreted with caution, taking prediction intervals into account. On the other hand, previous studies have also reported that, for example, higher training volume alone is not necessarily associated with improved cardiovascular health outcomes [108]. Instead, the relationship appears to be non‐linear and dose‐dependent: for example, blood pressure tends to decrease with increasing volume up to a point [104]. The current physical activity guidelines for Americans [2], as well as guidelines of WHO [4], recommend 2.5–5 h of weekly moderate‐intensity physical activity. The current results suggest that even a lower volume could be similarly beneficial, at least with the current setting involving interventions with structured endurance training of 0.8–7 weekly hours. While there was previously a minimum requirement of 10 min for the sustained duration of physical activity in the American guidelines [109], the updated guidelines have erased it [2]. Therefore, it would be important to examine the dose–response models of total accumulated physical activity at different intensity domains in terms of similar outcome variables as assessed in the intervention settings of this study.

### 4.4 Limitations of the Evidence

The current meta‐analysis with systematic review consisted of 50 studies. From these studies, only one standardized the exercise intensity according to LTs or VTs [61], although this approach could be regarded as the gold standard of exercise prescription [9]. Consequently, the exercise protocols were likely to produce different physiological strain among individuals, and it cannot be ruled out that some individuals were exercising at the heavy‐intensity domain instead of the intended moderate‐intensity domain. These limitations may have contributed to considerable heterogeneity in the results. Since it is a typical observation that there is large interindividual variation in the responses to standardized training [110], it is not surprising that the results also vary at the study level. However, the heterogeneity of the results is important to acknowledge when interpreting the results and their clinical relevance. Instead of relying on single pooled ES, prediction intervals provide supportive information for the possible range of single study results [111]. In the current analysis the certainty of evidence was rated as high for fitness‐related outcomes, whereas it varied from low to moderate for cardiometabolic variables, mainly due to high heterogeneity.

Regarding the reporting of training, there were inaccuracies in many studies involved: 24 studies did not report training adherence at any level, 14 reported adherence percentage at the session level, 12 reported the mean exercise intensity, while none reported the training intensity distribution. Since the adherence to training is a very significant contributor to training adaptations [112], it would be important to report the actual implemented training. Furthermore, monitoring and reporting habitual physical activity patterns from 24/7 data would provide relevant supportive information, as the background activity [113] or inactivity [114] could both confer specific effects on the training adaptations. The level of sufficient adherence remains an open and critical question. In the interventions included in this meta‐analysis that reported adherence, the range was 66%–100% and the mean was 93%. Some authors set the approval level as low as 66% [45], 70% [87], or 75% [46]. Since there is currently no clear definition of sufficient adherence, this needs further clarification in the future. Nevertheless, differences in the actual implementation of the training are also among the potential factors contributing to the between‐study heterogeneity in the results.

The direct assessment of VO_2max_ always requires the measurement of metabolic gases and ventilatory parameters, which would allow the assessment of VTs within the same test. Although a total of 37 studies reported VO_2max_ values in the current meta‐analysis, only five studies reported VT1, demonstrating the low exploitation of submaximal fitness parameters. In addition to exercise prescription, VT has been identified as a valuable outcome in cardiovascular disease risk classification [115] underscoring its relevance in exercise testing. Regarding VO_2max_, most studies reported only values in relation to body mass. While body mass could significantly change due to the intervention, absolute values would provide additional insights into actual cardiorespiratory adaptations [116]. Furthermore, detailed information on other maximum values (blood lactate, HR, respiratory exchange ratio) would be beneficial in training interventions to ensure comparable effort in all tests. This may help to mitigate the influence of learning effects, since no control tests were (reportedly) conducted in the interventions. The learning effect could hypothetically be one of the reasons for the greater magnitude of improvements in VO_2max_ in comparison to cardiometabolic variables. Another reason for smaller and more uncertain effects could relate to a considerable day‐to‐day variability of cardiometabolic variables. For example, the within‐subject day‐to‐day variation in total cholesterol, HDL, and LDL is reported to be between 6% and 10% [117], for glucose 13% [118], for triglycerides 23% [117], and for VO_2max_ 3% [118].

### 4.5 Implications of the Results and Recommendations for Future Research

The results of the current meta‐analysis can be considered encouraging because they demonstrated the beneficial effects of LIT on aerobic fitness and cardiometabolic health in the general working‐age adult population. Moreover, the improvements were observed with easily attainable training characteristics in terms of volume and intensity, and no definitive minimum intensity threshold for adaptations was detected across the main outcomes. Thus, for sedentary or untrained populations, LIT resembling the effort required in many activities of daily living may represent a feasible strategy to promote physical activity and support associated health‐ and fitness‐related adaptations. At the same time, the results also highlight that the intensity within the LIT domain should not be ignored, because it seems to influence fitness‐related adaptations. Most of the adaptations occurred quite rapidly, and longer interventions did not have further beneficial effects in any of the outcomes. Since progression is one of the main principles in exercise training [119], comparisons between progression methods (intensity, volume, frequency, combinations) should be examined in more detail. In parallel, it would be essential to address the contribution of individual 24/7 total physical activity and sedentary behavior to training‐induced adaptations. The relatively wide prediction intervals indicated high between‐study heterogeneity in the results. This probably relates to the actual uncertainty in responses despite standardized training, as well as aspects concerning methodological quality and the characteristics of study protocols. To enable more definitive conclusions, future research should prioritize high‐quality, comprehensively reported RCTs that standardize intensity prescription, measure and report a wider range of relevant physiological parameters, and explicitly track adherence.

### 4.6 Perspective

The present systematic review and meta‐analysis showed that LIT is an effective method to improve aerobic fitness and mitigate cardiometabolic risk factors among working‐age healthy sedentary or untrained individuals. Several meta‐analyses have compared HIT and LIT, and they have mostly reported that HIT is more effective in improving aerobic fitness in the short term [7, 21, 22], but in the long term, the intensity dependence is not as apparent [23]. Notably, the specific effects of intensity within the LIT domain have received less attention, although some studies have examined whether minimum effective exercise intensity could be determined for fitness‐ [12] or health‐related outcomes [13]. In the current analyses, no definitive minimum intensity for positive changes in fitness‐ or health‐related outcomes was found. However, the upper end of the LIT domain can be more beneficial for improving VO_2max_, whereas the lower intensity activities could still have comparable benefits on cardiometabolic variables. Since training adaptations were induced across a wide range of intervention durations, the results highlight the rapid occurrence of many adaptations in sedentary or untrained individuals, and simultaneously, the need for progressive training if further adaptations are desired. Given the relatively high heterogeneity of the results, notable differences in responses were observed across the studies, highlighting the importance of methodological quality (e.g., intensity prescription) in endurance training interventions.

## Funding

The authors have nothing to report.

## Ethics Statement

The authors have nothing to report.

## Consent

The authors have nothing to report.

## Conflicts of Interest

The authors declare no conflicts of interest.

## Supporting information


**Table S1:** Descriptive characteristics of all studies included in the meta‐analysis.
**Table S2:** Risk of bias analysis results within each study. Domains D1–D5: D1 = bias arising from the randomization process, D2 = bias due to deviations from intended interventions, D3 = bias due to missing outcome data, D4 = bias in measurement of the outcome, D5 = bias in selection of the reported results.
**Table S3:** Absolute pre‐values and changes in VO_2max_ (mL/kg/min).
**Table S4:** Absolute pre‐values and changes in VO_2max_ (L/min).
**Table S5:** Absolute pre‐values and changes in *P*_max_ (W).
**Table S6:** Absolute pre‐values and changes in VT1.
**Table S7:** Absolute pre‐values and changes in total cholesterol (all changed to mmol/L).
**Table S8:** Absolute pre‐values and changes in HDL cholesterol (all changed to mmol/L).
**Table S9:** Absolute pre‐values and changes in LDL cholesterol (all changed to mmol/L).
**Table S10:** Absolute pre‐values and changes in glucose (all changed to mmol/L).
**Table S11:** Absolute pre‐values and changes in triglycerides (all changed to mmol/L).
**Table S12:** Absolute pre‐values and changes in systolic blood pressure (mmHg).
**Table S13:** Absolute pre‐values and changes in diastolic blood pressure (mmHg).
**Table S14:** VO_2max_ (mL/kg/min) subgroup analysis. Effect size represents the effect of the low‐intensity training group compared with that of the control group.
**Table S15:** VO_2max_ (L/min) subgroup analysis. Effect size represents the effect of the low‐intensity training group compared with that of the control group.
**Table S16:** *P*_max_ (W) subgroup analysis. Effect size represents the effect of the low‐intensity training group compared with that of the control group.
**Table S17:** Total cholesterol subgroup analysis. Effect size represents the effect of the low‐intensity training group compared with that of the control group.
**Table S18:** HDL subgroup analysis. Effect size represents the effect of the low‐intensity training group compared with that of the control group.
**Table S19:** LDL subgroup analysis. Effect size represents the effect of the low‐intensity training group compared with that of the control group.
**Table S20:** Glucose subgroup analysis. Effect size represents the effect of the low‐intensity training group compared with that of the control group.
**Table S21:** Triglyceride subgroup analysis. Effect size represents the effect of the low‐intensity training group compared with that of the control group.
**Table S22:** Systolic blood pressure subgroup analysis. Effect size represents the effect of the low‐intensity training group compared with that of the control group.
**Table S23:** Diastolic blood pressure subgroup analysis. Effect size represents the effect of the low‐intensity training group compared with that of the control group.
**Table S24:** Results of leave‐one‐out analysis including the minimum and maximum ES and 95% CIs for each outcome.
**Table S25:** Results of leave one out analysis and 95% prediction interval (PI), including the minimum and maximum of 95% PIs for each outcome.
**Table S26:** The certainty of evidence according to GRADE.

## Data Availability

The data that support the findings of this study are available in the Supporting Information S1 of this article.
