# Supplementary material for: Effects of Low‐Intensity Endurance Training on Aerobic Fitness and Risk Factors of Cardiometabolic Health in Working‐Age Adults: A Systematic Review and Meta‐Analysis
Source: Scand J Med Sci Sports. 2026 Jan 16;36(1):e70208. doi: 10.1111/sms.70208 (PMC12810095; doi:10.1111/sms.70208)
Supplement: Supplementary file 1 — Table S1: Descriptive characteristics of all studies included in the meta‐analysis. Table S2: Risk of bias analysis results within each study. Domains D1–D5: D1 = bias arising from the randomization process, D2 = bias due to deviations from intended interventions, D3 = bias due to missing outcome data, D4 = bias in measurement of the outcome, D5 = bias in selection of the reported results. Table S3: Absolute pre‐values and changes in VO2max (mL/kg/min). Table S4: Absolute pre‐values and changes in VO2max (L/min). Table S5: Absolute pre‐values and changes in P max (W). Table S6: Absolute pre‐values and changes in VT1. Table S7: Absolute pre‐values and changes in total cholesterol (all changed to mmol/L). Table S8: Absolute pre‐values and changes in HDL cholesterol (all changed to mmol/L). Table S9: Absolute pre‐values and changes in LDL cholesterol (all changed to mmol/L). Table S10: Absolute pre‐values and changes in glucose (all changed to mmol/L). Table S11: Absolute pre‐values and changes in triglycerides (all changed to mmol/L). Table S12: Absolute pre‐values and changes in systolic blood pressure (mmHg). Table S13: Absolute pre‐values and changes in diastolic blood pressure (mmHg). Table S14: VO2max (mL/kg/min) subgroup analysis. Effect size represents the effect of the low‐intensity training group compared with that of the control group. Table S15: VO2max (L/min) subgroup analysis. Effect size represents the effect of the low‐intensity training group compared with that of the control group. Table S16: P max (W) subgroup analysis. Effect size represents the effect of the low‐intensity training group compared with that of the control group. Table S17: Total cholesterol subgroup analysis. Effect size represents the effect of the low‐intensity training group compared with that of the control group. Table S18: HDL subgroup analysis. Effect size represents the effect of the low‐intensity training group compared with that of the control group. Table S19: LDL subgroup [file SMS-36-e70208-s001.docx]

# Supplemental material

Supplemental materials for the article: “Effects of low-intensity endurance training on aerobic fitness and risk factors of cardiometabolic health in working-aged adults: A systematic review and meta-analysis”

By: Olli-Pekka Nuuttila, Pekka Matomäki, Jani Raitanen, Harri Sievänen, and Tommi Vasankari

In: Scandinavian Journal of Medicine & Science in Sport

| **Supplemental table 1.** Descriptive characteristics of all studies included in the meta-analysis. | | | | | | | | | | | |
| --- | --- | --- | --- | --- | --- | --- | --- | --- | --- | --- | --- |
| Study | Intervention  length | Training status | Outcomes | Sample size | Age | Intensity | Frequency | Duration | Volume/week | | Training mode |
| Asikainen et al. (2002) (1) | 24 weeks | Sedentary | VO_2max_ | CON: 20F  LIT1: 21F  LIT2: 16F  LIT3: 21F  LIT4: 38F | 56 ± 3.8 y  57 ± 3.8 y  55 ± 3.7 y  54 ± 3.5 y  55 ± 4.2 y | -  55%/ VO_2max_  45%/ VO_2max_  55%/ VO_2max_  45%/ VO_2max_ | -  5x/week  5x/week  5x/week  5x/week | -  300 kcal (54 ± 5.6 min)  300 kcal (65 ± 7.8 min)  200 kcal (38 ± 3.9 min)  200 kcal (46 ± 6.2 min) | -  4.5 h  5.4 h  3.2 h  3.8 h | -  Walking  Walking  Walking  Walking | |
| Baghaiee et al. (2016) (2) | 8 weeks | Untrained | HDL, TG, VO_2max_ | CON: 10M  LIT: 10M | 45.9 ± 3.2 y  44.9 ± 3.7 y | -  50–65%/HR_max_ | -  4x/week | -  40–50 min  (+10 min WU, and 5 min CD) | -  3.0 h | -  Treadmill | |
| Berger et al. (2006) (3) | 6 weeks | Sedentary | GET, VO_2max_ | CON: 7  (4F, 3M)  LIT: 8  (5F, 3M) | 24 ± 5 y  24 ± 5 y | -  60%/ VO_2max_ | -  3–4x/week | -  30 min | -  1.9 h | -  Cycling | |
| Blond et al. (2019) (4) | 6 months | Physically inactive | Gl, VO_2max_ | CON: 18 (9F, 9M)  LIT: 39 (21F, 18M) | 35 ± 7 y  32 ± 7 y | -  50%/ VO_2max_ | -  2–5x/week | -  F: 320 kcal  M: 420 kcal  Avg: 52 (46;61) min | -  4.2 h | -  Walking, running, rowing, cross trainer, stationary cycling | |
| Camargo et al. (2007) (5) | 12 weeks | Sedentary | VO_2max_, VT1 | CON: 7M  LIT: 6M | 29 ± 4 y  30 ± 4 y | -  70%/HR_max_ | -  3x/week | -  25 min (+ 5 min WU, and 5 min CD) | -  1.3 h | -  Treadmill | |
|  |  |  |  |  |  |  |  |  |  |  | |
| Eguchi et al. (2012) (6) | 12 weeks | General population | Gl, DBP, HDL, LDL, SBP, TC | CON: 10F  LIT: 8F | 52.9 ± 9.7 y  50.7 ± 6.5 y | -  50%/ VO_2max_ | -  3x/week | -  30 min | -  1.5 h | -  Bicycle ergometer | |
| Gonçalves et al. (2022) (7) | 12 weeks | Sedentary | Gl, DBP, HDL, LDL, SBP, TC, TG, | CON: 8M  LIT: 11M | 41.8 ± 3.0 y  39.3 ± 3.0 y | -  50–60%/HRR | -  3/week | -  30–50 min | -  2.3 h | -  Treadmill and stationary bike | |
| Gormley et al. (2008) (8) | 6 weeks | Less than 3 h cycling/week | DBP, SBP, VO_2max_ | CON: 13  (8F, 5M)  LIT: 14  (9F, 5M) | 22 ± 3.0 y  23 ± 4.0 y | -  50%/HRR | -  3–4x/week | -  30–60 min | -  3.3 h | -  Bicycle ergometer | |
| Gossard et al. (1986) (9) | 12 wk | No regular vigorous PA | Economy, VO_2max_ | CON: 20M  LIT: 20M | 40-60 y  40-60 y | -  60–72%/HR_max_ | -  5x/week | -  At least 300 kcal (52.2 ± 5 min) | -  4.3 h | -  Walking/running | |
| Grieco et al. (2013) (10) | 6 weeks | Less than 3 h cycling/week | Gl, HDL, TC, VO_2max_ | CON: 7  (F/M NA)  LIT: 10  (F/M NA) | 22.2 ± 3.9 y (all groups) | -  50%/HRR | -  3–4x/week | -  30–60 min | -  3.3 h | -  Bicycle ergometer | |
| Hiruntrakul et al. (2010) (11) | 12 weeks | Sedentary | HDL | CON: 18M  LIT: 19M | 21 ± 2 y  20 ± 1 y | -  60%/ VO_2max_ | -  1x/week | -  50 min (+5 min WU, and 5 min CD) | -  0.8 h | -  Bicycle ergometer | |
| Hu et al. (2021) (12) | 12 weeks | Sedentary | VO_2max_ | CON: 15F  LIT: 15F | 20.9 ± 1.1 y  20.9 ± 1.4 y | -  60%/ VO_2max_ | -  3x/week | -  200-300 Kcal  Avg: 65.3 min | -  3.3 h | -  Cycling ergometer | |
| Huang et al. (2019a) (13) | 6 weeks | Sedentary | VO_2max_, VT1 | CON: 18M  LIT: 18M | 22.0 ± 2.1 y  21.9 ± 2.5 y | -  60%/ VO_2max_ | -  5x/week | -  30 min (+3 min WU, and 3 min CD) | -  2.5 h | -  Cycling ergometer | |
| Huang et al. (2019b) (14) | 6 weeks | Sedentary | DBP, SBP, VO_2max_, VT1 | CON: 15M  LIT: 15M | 21.2 ± 3.1 y  22.1 ± 2.7 y | -  60%/HRR | -  5x/week | -  30 min (+3 min WU, and 3 min CD) | -  2.5 h | -  Bicycle ergometer | |
| Huttunen et al. (1979) (15) | 8 weeks | Physically rather inactive during the year preceding study | HDL, LDL, TC, TG | CON: 46M  LIT: 44M | 40-45 y  40-45 y | -  40%/HRR | -  3x/week | -  30 min  (+15 min WU, 10 min CD) | 1.5 h | -  Walking, jogging, swimming, skiing or cycling | |
| Kong et al. (2021) (16) | 12 weeks | Sedentary | VO_2max_ | CON: 17F  LIT: 19F | 21.1 ± 1.4 y  20.8 ± 1.5 y | -  60%/ VO_2max_ | -  3–4x/week | -  200–300 Kcal  Avg: 63 min (+10 min WU, and 5 min CD) | -  3.9 h | -  Cycling ergometer | |
| Lee et al. (2012)  (17) | 14 weeks | Untrained | HDL, LDL, TC, TG, VO_2max_ | CON: 7F  LIT: 8F | 38.3 ± 4.9 y  41.6 ± 4.5 y | -  50%/ VO_2max_ | -  3–5x/week | -  4.5 METsh/session (72.9 ± 2.2 min) | -  4.9 h | -  Running/walking | |
| Lemura et al. (2000) (18) | 8 weeks | Sedentary | HDL, LDL, TC, TG, VO_2max_ | CON: 12F  LIT: 10F | 20 ± 1 y  21 ± 2 y | -  70–75%/HR_max_ | -  3x/week | -  30 min (+10 min WU, and 10 min CD) | -  1.5 h | -  Cycling ergometer, rowing ergometer or treadmill | |
| Lucibello et al. (2019) (19) | 9 weeks | Untrained | VO_2max_ | CON: 20  (15F, 5M)  LIT: 22  (19F, 3M) | 19.2 ± 1.4  19.7 ± 1.5 | -  70–75%/HR_max_ | -  3x/week | -  27.5 min (+3 min WU, and 2.5 min CD) | -  1.4 h | -  Stationary cycling | |
| McDonald et al. (1993) (20) | 10 weeks | Sedentary | DBP, SBP, VO_2max_ | CON: 4F  LIT: 9M | 22-34 y  22-34 y | -  60%/ VO_2max_ | -  3x/week | -  60 min | -  3 h | -  Cycle ergometer | |
| McDowell et al. (1992) (21) | 10 weeks | Novice runners | VO_2max_ | CON: 5M  LIT: 8M | 20.3 ± 2.3 y  23.3 ± 3.9 y | -  70%/HR_max_ | -  3x/week | -  20 min | -  1 h | -  Running | |
| Meyer et al. (2006) (22) | 12 weeks | Untrained | LT, VO_2max_ | CON: 13  (6F, 7M)  LIT: 13  (7F, 6M) | 46 ± 7 y  42 ± 7 y | -  71%/HR_max_ | -  5x/week | -  35.4 min (+10 min WU) | -  3.0 h | -  Walking/running | |
| Mousavi et al. (2023) (23) | 8 weeks | Untrained | HDL, LDL, TC, TG | CON: 9M  LIT: 10M | 27.7 ± 2.5 y  28.0 ± 2.7 y | -  55–70%/HR_max_ | -  3x/week | -  20–35 min (+10 min WU, and 10 min CD) | -  1.3 h | -  Walking/running | |
| Murphy et al. (2006) (24) | 8 weeks | Did not meet PA recommendations | DBP, HDL, LDL SBP, TC, TG | CON: 12  (8F, 6M)  LIT: 21  (16F, 7M) | 40.8 ± 10 y  41.4 ± 7.5 y | -  62.1%/HR_max_ | -  2x/week | -  25-45 min | -  1.4 h | -  Walking outdoors | |
| Poon et al. (2022) (25) | 16 weeks | Inactive | Gl, DBP, HDL, LDL, SBP, TC, TG, VO_2max_ | CON: 10M  LIT: 10M | 40.1 ± 3.6 y  44.8 ± 6.5 y | -  65-70%/HR_max_ | -  3x/week | -  20–40 min (+3 min WU, and 3 min CD) | -  1.8 h | -  Walking outdoors, treadmill | |
| Rogers et al. (1996) (26) | 12 weeks | Untrained | DBP, SBP, VO_2max_ | CON: 5  (F/M NA)  LIT: 6  (F/M NA) | 40.2 ± 6.3 y  44.8 ± 7.6 y | -  45%/ VO_2max_ | -  3x/week | -  45 min | -  2.3 h | -  Treadmill walking/running | |
| Roxburgh et al. (2014) (27) | 12 weeks | Sedentary | VO_2max_ | CON: 7  (F/M NA)  LIT: 6  (F/M NA) | 34.4 ± 4.7 y  36.5 ± 9.2 y | -  45–60%/HRR | -  5x/week | -  30 min | -  2.5 h | -  Treadmill walking + cycling | |
| Seifert et al. (2009) (28) | 3 months | Sedentary | VO_2max_ | CON: 8M  LIT: 10M | 32 ± 6 y  30 ± 5 y | -  70%/HR_max_ | -  7x/week | -  2500 KJ (∼60 min) | -  7 h | -  Primarily cycling, also alternative modes such as running and rowing allowed | |
| Shabani et al. (2018) (29) | 8 weeks | Untrained | VO_2max_ | CON: 6F  LIT: 9F | 25.5 ± 4.8 y  24.7 ± 2.3 y | -  55–75%/HR_max_ | -  3x/week | -  50 min (+10 min WU, and 5 min CD) | -  2.5 h | -  Rhythmic aerobic step training and running | |
| Shaw et al. (2009a,b) (30,31) | 16 weeks | Untrained | LDL^b^, VO_2max_^a^ | CON: 12M  LIT: 12M | 25 ± 2.4 y  25 ± 5.6 y | -  60–75%/HR_max_ | -  3x/week | -  45 min (+5 min WU, and 5 min CD) | -  2.3 h | -  Treadmill, rower, stepper or cycle ergometer | |
| Sittiwicheanwong et al. (2007) (32) | 12 weeks | Sedentary | HDL, LDL, TC, TG | CON: 15F  LIT: 20F | 46.3 ± 5.0 y  47.0 ± 5.4 y | -  60%/ VO_2max_ | -  3x/week | -  25 min (+10 min WU, and 10 min CD) | -  1.3 h | -  Cycling ergometer | |
| Sloan et al. (2018) (33) | 12 weeks | Sedentary | VO_2max_ | CON: 58  (F/M NA)  LIT: 45  (F/M NA) | 31.4 ± 6.2 y  31.2 ± 5.7 y | -  55–75%/HR_max_ | -  4x/week | -  30–40 min (+10–15 min WU, and 10–15 min CD) | -  2.3 h | -  Permitted to select from a series of aerobic activities | |
| Soori et al. (2018) (34) | 10 weeks | Sedentary | HDL, LDL, TL, TG | CON: 8F  LIT: 8F | 45-60 y  45-60 y | -  40–60%/HR_max_ | -  3x/week | -  45 min | -  2.3 h | -  Swimming | |
| Stein et al. (1990) (35) | 12 weeks | Not specified | HDL, LDL, TC, TG, VO_2max_ | CON: 10M  LIT1: 13M  LIT2: 14M | 44 ± 8 y  (all groups) | -  65%/HR_max_  75%/HR_max_ | -  3x/week  3x/week | -  30 min  30 min | -  1.5 h  1.5 h | -  Cycling ergometer  Cycling ergometer | |
| Stein et al. (1992) (36) | 8 weeks | Sedentary | VO_2max_ | CON: 14M  LIT: 19M | 45.0 ± 6.1 y  46.2 ± 6.1 y | -  60%/HRR | -  3x/week | -  20–30 min | -  1.4 h | -  Walking, running, cycling ergometer | |
| Suter et al. (1994) (37) | 6 months | Sedentary | HDL, TC, TG, VO_2max_ | CON: 28M  LIT: 19M | 42.1 ± 7.6 y  42.2 ± 7.4 y | -  50%/ VO_2max_ | -  6x/week | -  30 min | -  3.0 h | -  Walking | |
| Tan et al. (2016)  (38) | 10 weeks | Not engaged in regular exercise over the past 2 years | Gl, HDL, TC, TG | CON: 11F  LIT: 15F | 49.7 ± 7.9 y  50.7 ± 5.5 y | -  62%/HR_max_ | -  5x/week | -  40 min | -  3.3 h | -  Walking or running | |
| Thompson et al. (2009) (39) | 12 weeks | Sedentary | DBP, HDL, SBP, TC, TG | CON: 21M  LIT: 20M | 54 ± 5 y  52 ± 4 y | -  50–55%/HR_max_ | -  3–4x/week | -  30–45 min | -  2.3 h | -  Not specified | |
| Tsai et al. (2002a) (40) | 12 weeks | Not engaged in an exercise program prior to the study | DBP, HDL, LDL, SBP, TC, TG | CON: 11  (6F, 5M)  LIT: 12  (5F, 7M) | 46.2 ± 5.6 y  49.6 ± 9.3 | -  60–70%/HR_max_ | -  3x/week | -  30 min (+10 min WU, and 10 min CD) | -  1.5 h | -  Treadmill | |
| Tsai et al. (2002b) (41) | 12 weeks | Not currently performing regular aerobic exercise | DBP, HDL, LDL, SBP, TC, TG | CON: 20  (9F, 11M)  LIT: 22 (10F, 12M) | 36.6 ± 7.4 y  45.5 ± 9.5 y | -  60–70%/HR_max_ | -  3x/week | -  30 min (+10 min WU, and 10 min CD) | -  1.5 h | -  Treadmill | |
| Tseng et al. (2013) (42) | 12 weeks | Not specified (obese) | Gl, DBP, HDL, SBP, TG | CON: 10M  LIT: 10M | 22.3 ± 1.0 y  22.1 ± 1.1 y | -  50–70%/HR_max_ | -  5x/week | -  60 min (+10 min WU, and 10 min CD) | -  5 h | -  Treadmill | |
| van Aggel-Leijssen et al. (2001) (43) | 12 weeks | Did not spend more than 2 h/week in sports activities and had no physically demanding job | Gl, VO_2max_ | CON: 8M  LIT1: 7M  LIT2: 6M | 43.3 ± 5.4 y  42.9 ± 6.6 y  42.7 ± 4.9 y | -  40%/VO_2max_  40%/ VO_2max_ | -  3x/week  3x/week | -  5 Kcal/FFMkg,∼57 min  5 Kcal/FFMkg,∼57 min | -  2.9 h  2.9 h | -  Cycling ergometer  Cycling ergometer | |
| Wang et al. (1995) (44) | 8 weeks | Sedentary | DBP, SBP, VO_2max_ | CON: 12M  LIT: 11M | 21.7 ± 2.1 y  21.0 ± 2.3 y | -  60%/ VO_2max_ | -  5x/week | -  30 min | -  2.5 h | -  Bicycle ergometer | |
| Wang et al. (1997) (45) | Two consecutive menstrual cycles | Sedentary | DBP, SBP, VO_2max_ | CON: 8F  LIT: 8F | 22.8 ± 3.7 y  21.3 ± 2.3 y | -  50%/ VO_2max_ | -  5x/week | -  30 min | -  2.5 h | -  Bicycle ergometer | |
| Wang et al. (2005) (46) | 8 weeks | Sedentary | DBP, SBP, VO_2max_ | CON: 15M  LIT: 15M | 24.7 ± 8.9 y  23.5 ± 6.2 y | -  60%/ VO_2max_ | -  5x/week | -  30 min | -  2.5 h | -  Bicycle ergometer | |
| Wang et al. (2011) (47) | 4 weeks | Sedentary | VO_2max_ | CON: 10M  LIT: 10M | 22.9 ± 1.3 y  21.5 ± 2.2 y | -  50%/ VO_2max_ | -  5x/week | -  30 min | -  2.5 h | -  Bicycle ergometer | |
| Werner et al. (2019) (48) | 6 months | Sedentary | Gl, DBP, LT, SBP, TC, VO_2max_ | CON: 35  (23F, 12M)  LIT: 26  (17F, 9M) | 50.2 ± 7.4 y  49.5 ± 7.0 y | -  60%/HRR | -  3x/week | -  45 min | -  2.3 h | -  Walking/running | |
| Wu et al. (2017) (49) | 6 weeks | Sedentary | DBP, SBP, VO_2max_, VT | CON: 15M  LIT: 15M | 21.9 ± 2.7 y  22.1 ± 1.9 y | -  60%/ VO_2max_ | -  5x/week | -  30 min | -  2.5 h | -  Cycling ergometer | |
| Zhang et al. (2015) (50) | 12 weeks | No regular physical training | Gl, TC, TG, VO_2max_ | CON: 11F  LIT: 12F | 20.9 ± 1.0 y  20.6 ± 1.2 y | -  60–70%/HR_max_ | -  4x/week | -  33 min (+10 min WU, and 5 min CD) | -  2.2 h | -  Running | |
|  |  |  |  |  |  |  |  |  |  |  | |

Avg = average; CD = cool-down; CON = control group; DBP = diastolic blood pressure; Gl = Glucose; F = female participants; GET = gas exchange threshold; HDL = high-density lipoprotein; HRmax = maximum heart rate; HRR = heart rate reserve; LIT = low-intensity training; LDL = low-density lipoprotein; LT = lactate threshold; M = male participants; NA = not available; SBP = systolic blood pressure; TC = total cholesterol; TG = Triglycerides; VO_2max_ = maximum oxygen uptake; VT = ventilatory threshold; WU = warm-up.

| **Supplemental table 2.** Risk of bias analysis results within each study. Domains D1–D5: D1 = bias arising from the randomization process, D2 = bias due to deviations from intended interventions, D3 = bias due to missing outcome data, D4 = bias in measurement of the outcome, D5 = bias in selection of the reported results. | | | | | |
| --- | --- | --- | --- | --- | --- |
| Study | D1 | D2 | D3 | D4 | D5 |
| (1) Asikainen et al. (2002) | + | - | + | + | - |
| (2) Baghaiee et al. (2016) | - | - | + | - | - |
| (3) Berger et al. (2006) | - | + | + | + | - |
| (4) Blond et al. (2019) | + | + | + | - | + |
| (5) Camargo et al. (2007) | - | + | + | + | - |
| (6) Eguchi et al. (2012) | - | - | - | - | - |
| (7) Gonçalves et al. (2022) | - | - | - | + | - |
| (8) Gormley et al. (2008) | - | + | + | + | - |
| (9) Gossard et al. (1986) | - | + | + | + | - |
| (10) Grieco et al. (2013) | - | - | + | + | - |
| (11) Hiruntrakul et al. (2010) | + | - | + | + | - |
| (12) Hu et al. (2021) | + | + | + | + | - |
| (13) Huang et al. (2019a) | - | + | + | + | - |
| (14) Huang et al. (2019b) | - | + | + | + | - |
| (15) Huttunen et al. (1979) | + | - | + | + | - |
| (16) Kong et al. (2021) | - | + | + | + | - |
| (17) Lee et al. (2012) | - | - | + | + | + |
| (18) Lemura et al. (2000) | - | - | + | - | - |
| (19) Lucibello et al. (2019) | + | + | + | + | - |
| (20) McDonald et al. (1993) | - | - | - | - | - |
| (21) McDowell et al. (1992) | - | - | + | + | - |
| (51) Meyer et al. (2006) | + | + | + | + | - |
| (23) Mousavi et al. (2023) | + | - | + | - | + |
| (24) Murphy et al. (2006) | - | - | - | + | - |
| (25) Poon et al. (2022) | + | - | + | + | - |
| (26) Rogers et al. (1996) | - | - | + | + | - |
| (27) Roxburgh et al. (2014) | - | - | + | + | - |
| (28) Seifert et al. (2009) | - | - | - | + | - |
| (29) Shabani et al. (2018) | - | - | - | + | - |
| (30) Shaw et al. (2009a) | - | - | - | - | - |
| (31) Shaw et al. (2009b) | - | - | - | - | - |
| (32) Sittiwicheanwong et al. (2007) | - | - | - | + | - |
| (33) Sloan et al. (2018) | + | - | - | - | + |
| (34) Soori et al. (2018) | - | - | + | + | - |
| (35) Stein et al. (1990) | - | + | + | + | - |
| (36) Stein et al. (1992) | - | - | + | + | - |
| (37) Suter et al. (1994) | - | - | + | + | - |
| (38) Tan et al. (2016) | - | - | - | + | - |
| (39) Thompson et al. (2009) | + | + | + | + | - |
| (40) Tsai et al. (2002a) | - | + | + | + | - |
| (41) Tsai et al. (2002b) | - | + | + | + | - |
| (42) Tseng et al. (2013) | - | - | + | + | - |
| (43) van Aggel-Leijssen et al. (2001) | - | + | + | + | - |
| (44) Wang et al. (1995) | - | - | + | + | - |
| (45) Wang et al. (1997) | - | - | + | + | - |
| (46) Wang et al. (2005) | - | - | + | + | - |
| (47) Wang et al. (2011) | - | + | + | + | - |
| (48) Werner et al. (2019) | + | - | - | + | + |
| (49) Wu et al. (2017) | - | + | + | + | - |
| (50) Zhang et al. (2015) | - | + | + | + | - |

**Supplemental table 3.** Absolute pre-values and changes in VO_2max_ (ml/kg/min).

| Study | Pre-  control | Change control | Pre-  LIT | Change  LIT | Change  LIT vs. control |
| --- | --- | --- | --- | --- | --- |
| Asikainen et al. 2002 1 | 29.2 ± 3.7 | -1.7 | 30.3 ± 5.1 | 1.0 | 2.7 |
| Asikainen et al. 2002 2 | 29.2 ± 3.7 | -1.7 | 30.8 ± 4.2 | 0.6 | 2.3 |
| Asikainen et al. 2002 3 | 29.2 ± 3.7 | -1.7 | 29.9 ± 4.1 | 0.5 | 2.2 |
| Asikainen et al. 2002 4 | 29.2 ± 3.7 | -1.7 | 30.2 ± 2.8 | 0.3 | 2.0 |
| Baghaiee et al. 2016 | 32.1 ± 4.2 | -1.6 | 33.5 ± 5.4 | 5.1 | 6.8 |
| Berger et al. 2006 | 34.5 ± 5.9 | 0.9 | 33.2 ± 4.0 | 7.1 | 6.2 |
| Blond et al. 2019 | 29.3 ± 5.9 | -0.8 | 28.8 ± 5.2 | 3.1 | 3.9 |
| Camargo et al. 2007 | 38.0 ± 2.0 | -0.8 | 38.1 ± 2.0 | 4.4 | 5.2 |
| Gormley et al. 2008 | 37.7 ± 8.7 | 0.7 | 35.3 ± 7.9 | 3.4 | 2.7 |
| Gossard et al. 1986 | 30.9 ± 4.8 | 0.2 | 33.3 ± 3.5 | 2.7 | 2.5 |
| Grieco et al. 2013 | 37.8 ± 7.8 | 1.8 | 38.2 ± 11.4 | 2.2 | 0.4 |
| Hu et al. 2021 | 28.8 ± 3.6 | -1.2 | 30.6 ± 3.5 | 7.7 | 8.9 |
| Huang et al. 2019a | 34.4 ± 4.7 | 0.6 | 36.1 ± 5.5 | 5.8 | 5.2 |
| Huang et al. 2019b | 34.0 ± 6.2 | -0.1 | 35.3 ± 8.1 | 5.9 | 6.0 |
| Kong et al. 2021 | 29.1 ± 3.4 | -1.1 | 27.8 ± 3.2 | 3.5 | 4.6 |
| Lee et al. 2012 | 29.3 ± 1.5 | -1.9 | 26.3 ± 2.8 | 5.3 | 7.2 |
| Lemura et al. 2000 | 33.3 ± 2.7 | 0.8 | 33.5 ± 4.4 | 4.5 | 3.7 |
| Lucibello et al. 2019 | 28.0 ± 6.8 | -0.1 | 28.5 ± 6.8 | 2.2 | 2.3 |
| McDonald et al. 1993 | 42.8 ± 7.6 | -3.8 | 45.6 ± 7.5 | 4.4 | 8.2 |
| McDowell et al. 1992 | 54.2 ± 2.9 | -1.0 | 47.8 ± 3.2 | 2.9 | 3.9 |
| Meyer et al. 2006 | 35.7 ± 9.1 | -1.0 | 36.0 ± 6.1 | 1.7 | 2.7 |
| Poon et al. 2022 | 36.4 ± 7.2 | -0.3 | 34.9 ± 5.0 | 4.5 | 4.8 |
| Rogers et al. 1996 | 28.3 ± 5.6 | -0.3 | 27.3 ± 3.9 | 1.0 | 1.3 |
| Roxburgh et al. 2014 | 30.0 ± 4.6 | -1.7 | 33.2 ± 4.0 | 1.3 | 3.0 |
| Shabani et al. 2018 | 37.2 ± 2.0 | -1.2 | 33.2 ± 2.1 | 4.4 | 5.6 |
| Shaw et al. 2009 | 27.4 ± 3.6 | -0.9 | 29.8 ± 4.8 | 10.2 | 11.1 |
| Sloan et al. 2018 | 31.0 ± 6.4 | -0.9 | 30.4 ± 6.9 | 4.4 | 5.2 |
| Stein et al. 1990 1 | 30.4 ± 6.1 | 1.3 | 28.4 ± 4.9 | 3.1 | 1.8 |
| Stein et al. 1990 2 | 30.4 ± 6.1 | 1.3 | 30.3 ± 5.0 | 5.6 | 4.3 |
| Suter et al. 1994 | 36.3 ± 4.8 | -1.2 | 35.3 ± 5.9 | 2.5 | 3.7 |
| Wang et al. 1995 | 34.1 ± 5.5 | -0.7 | 34.6 ± 7.5 | 7.6 | 8.3 |
| Wang et al. 1997 | 25.2 ± 2.8 | 0.1 | 26.1 ± 3.1 | 10.5 | 10.4 |
| Wang et al. 2005 | 30.2 ± 10.5 | 2.6 | 29.8 ± 4.3 | 10.1 | 7.5 |
| Wang et al. 2011 | 43.5 ± 11.4 | 1.3 | 44.1 ± 7.9 | 4.5 | 3.2 |
| Werner et al. 2019 | 35.1 ± 5.3 | -0.9 | 35.3 ± 6.3 | 2.6 | 3.5 |
| Wu et al. 2017 | 31.4 ± 2.3 | 0.0 | 32.2 ± 2.3 | 4.0 | 4.0 |
| Zhang et al. 2015 | 30.3 ± 3.6 | -1.7 | 33.0 ± 3.3 | 4.6 | 6.3 |
| **Pooled mean values** | **32.5 ± 5.8** | **-0.5 ± 4.2** | **32.5 ± 5.5** | **3.9 ± 4.2** | **4.5 ± 2.4** |

LIT = Low-intensity training

**Supplemental table 4.** Absolute pre-values and changes in VO_2max_ (l/min).

| Study | Pre-  control | Change control | Pre-  LIT | Change  LIT | Change  LIT vs. control |
| --- | --- | --- | --- | --- | --- |
| Berger 2006 | 2.51 ±0.65 | 0.06 | 2.34 ± 0.50 | 0.47 | 0.41 |
| Hu 2021 | 1.90 ± 0.20 | 0.00 | 2.10 ± 0.30 | 0.40 | 0.40 |
| Roxburgh 2014 | 2.40 ± 0.40 | -0.10 | 3.00 ± 0.90 | 0.10 | 0.20 |
| Seifert 2009 | 3.40 ± 0.20 | 0.00 | 3.40 ± 0.40 | 0.70 | 0.70 |
| Stein 1992 | 2.90 ± 0.40 | 0.00 | 2.90 ± 0.40 | 0.40 | 0.40 |
| van Aggel-Leijssen 2001 1 | 2.94 ± 0.44 | 0.08 | 3.19± 0.53 | 0.37 | 0.29 |
| van Aggel-Leijssen 2001 2 | 2.94 ± 0.44 | 0.08 | 3.22± 0.42 | 0.07 | -0.01 |
| **Pooled mean values** | **2.610 ± 0.38** | **0.01 ± 0.31** | **2.80 ± 0.47** | **0.39 ± 0.41** | **0.39 ± 0.19** |

**Supplemental table 5.** Absolute pre-values and changes in P_max_ (W).

| Study | Pre-  control | Change control | Pre-  LIT | Change  LIT | Change  LIT vs. control |
| --- | --- | --- | --- | --- | --- |
| Berger 2006 | 215 ± 57 | 2 | 195 ± 44 | 34 | 32 |
| Huang 2019b | 200 ± 35 | 2 | 190 ± 31 | 31 | 29 |
| Huang 2019 | 202 ± 34 | -1 | 190 ± 25 | 32 | 33 |
| Seifert 2009 | 289 ± 24 | 4 | 302 ± 45 | 63 | 59 |
| Wang 1995 | 169 ± 21 | -2 | 181 ± 39 | 43 | 45 |
| Wang 1997 | 93 ± 11 | 3 | 101 ± 11 | 34 | 30 |
| Wang 2005 | 194 ± 43 | 2 | 195 ± 27 | 44 | 42 |
| **Pooled mean values** | **192 ± 33** | **1 ± 24** | **193 ± 30** | **36 ± 22** | **35 ± 11** |

**Supplemental table 6.** Absolute pre-values and changes in VT1.

| Study | Pre-  control | Change control | Pre-  LIT | Change  LIT | Change  LIT vs. control |
| --- | --- | --- | --- | --- | --- |
| *VO_2_ (l/min)* |  |  |  |  |  |
| Berger et al. 2006 | 1.29 ±0.36 | 0.08 | 1.10 ± 0.15 | 0.47 | 0.39 |
| *VO_2_ (ml/kg/min)* |  |  |  |  |  |
| Camargo et al. 2007 | 17.8 ± 1.6 | -0.1 | 17.4 ± 2.2 | 2.3 | 2.4 |
| Huang et al. 2019a | 20.8 ± 6.8 | 0.4 | 21.0 ± 4.2 | 2.6 | 2.2 |
| Huang et al. 2019b | 20.1 ± 6.6 | 1.5 | 21.2 ± 7.0 | 2.6 | 1.1 |
| Meyer et al. 2006 | 26.0 ± 5.2 | 0.6 | 24.4 ± 4.4 | 0.8 | 0.2 |
| Wu et al. 2017 | 16.9 ± 3.5 | 0.1 | 17.5 ± 2.7 | 3.7 | 3.6 |
| **Pooled mean values** | **20.5 ± 5.5** | **0.6 ± 4.9** | **20.6 ± 4.9** | **2.5 ± 3.6** | **1.9 ± 1.2** |

LIT = Low-intensity training

**Supplemental table 7.** Absolute pre-values and changes in total cholesterol (all changed to mmol/l).

| Study | Pre-  control | Change control | Pre-  LIT | Change  LIT | Change  LIT vs.  control | Serum (s)  Plasma (p)  Not reported (nr) |
| --- | --- | --- | --- | --- | --- | --- |
| Eguchi et al. 2012 | 5.67 ± 0.79 | -0.02 | 5.83 ± 0.55 | -0.29 | -0.26 | nr |
| Goncalves et al. 2022 | 4.84 ± 0.26 | -0.30 | 5.41 ± 0.36 | -0.23 | 0.07 | s |
| Grieco et al. 2013 | 4.10 ± 0.90 | -0.10 | 4.30 ± 0.60 | -0.10 | 0.20 | p? |
| Huttunen et al. 1979 | 6.82 ± 0.86 | -0.26 | 6.76 ± 1.12 | -0.34 | -0.08 | s |
| Lee et al. 2012 | 4.91 ± 0.55 | -0.37 | 4.81 ± 0.56 | -0.04 | 0.34 | s |
| Lemura et al. 2000 | 5.00 ± 0.40 | 0.00 | 5.10 ± 0.40 | -0.20 | -0.20 | p? |
| Mousavi et al. 2023 | 3.86 ± 0.19 | -0.01 | 3.87 ± 0.18 | -0.33 | -0.32 | s |
| Murphy et al. 2006 | 5.70 ± 1.30 | 0.4 | 5.7 ± 1.10 | 0.30 | -0.10 | s |
| Poon et al. 2022 | 5.60 ± 0.95 | -0.22 | 5.78 ± 0.58 | -0.25 | -0.03 | nr |
| Sittiwicheanwong et al. 2007 | 5.69 ± 0.43 | -0.07 | 5.77 ± 0.49 | -0.22 | -0.14 | p |
| Soori et al. 2017 | 5.61 ± 0.65 | 0.54 | 6.26 ± 0.59 | -0.70 | -1.24 | s |
| Stein et al. 1990 1 | 5.08 ± 1.37 | 0.24 | 5.55 ± 1.51 | -0.17 | -0.41 | p |
| Stein et al. 1990 2 | 5.08 ± 1.37 | 0.24 | 5.61 ± 1.07 | -0.28 | -0.52 | p |
| Suter et al. 1994 | 5.55 ± 1.14 | 0.15 | 5.78 ± 0.92 | 0.16 | 0.01 | s |
| Tan et al. 2016 | 4.43 ± 0.56 | -0.02 | 4.35 ± 0.55 | -0.11 | -0.09 | nr |
| Thompson et al. 2009 | 5.18 ± 0.76 | -0.26 | 5.31 ± 1.10 | 0.07 | 0.33 | p |
| Tsai et al. 2002a | 5.27 ± 0.75 | 0.09 | 5.20 ± 0.73 | -0.35 | -0.44 | nr |
| Tsai et al. 2002b | 5.09 ± 0.39 | 0.07 | 4.93 ± 0.46 | -0.30 | -0.37 | p |
| Werner et al. 2019 | 5.48 ± 1.03 | 0.10 | 5.79 ± 1.11 | -0.28 | -0.39 | nr |
| Zhang et al. 2015 | 3.36 ± 0.42 | 0.09 | 4.34 ± 0.82 | -0.53 | -0.62 | s |
| **Pooled mean values** | **5.4 ± 0.83** | **-0.02 ± 0.75** | **5.51 ± 0.89** | **-0.18 ± 0.71** | **-0.19  ± 0.29** | **p: 7**  **s: 8 nr: 5** |

*? =* Not totally unequivocally reported, LIT = Low-intensity training

**Supplemental table 8.** Absolute pre-values and changes in HDL cholesterol (all changed to mmol/l).

| Study | Pre-  control | Change control | Pre-  LIT | Change  LIT | Change  LIT vs.  control | Plasma (p) Serum (s)  Not reported (nr) |
| --- | --- | --- | --- | --- | --- | --- |
| Baghaiee et al. 2016 | 0.93 ± 0.06 | -0.02 | 0.98 ± 0.09 | 0.31 | 0.33 | nr |
| Eguchi et al. 2012 | 1.67 ± 0.31 | -0.06 | 1.61 ± 0.43 | -0.09 | -0.04 | nr |
| Goncalves et al. 2022 | 1.17 ± 0.10 | 0.04 | 1.20 ± 0.05 | 0.10 | 0.06 | s |
| Grieco et al. 2013 | 1.60 ± 0.50 | -0.10 | 1.30 ± 0.30 | 0.0 | 0.10 | p? |
| Hiruntrakul et al. 2010 | 1.18 ± 0.20 | 0.02 | 1.16 ± 0.33 | 0.04 | 0.01 | nr |
| Huttunen et al. 1979 | 1.24 ± 0.26 | -0.01 | 1.26 ± 0.27 | 0.06 | 0.07 | s |
| Lee et al. 2012 | 1.37 ± 0.26 | -0.03 | 1.41 ± 0.26 | 0.06 | 0.09 | s |
| Lemura et al. 2000 | 1.40 ± 0.10 | 0.10 | 1.40 ± 0.10 | 0.10 | 0.00 | p? |
| Mousavi et al. 2023 | 0.98 ± 0.27 | 0.0 | 0.99 ± 0.06 | 0.10 | 0.10 | s |
| Murphy et al. 2006 | 1.10 ± 0.30 | 0.30 | 1.10 ± 0.30 | 0.20 | -0.10 | s |
| Poon et al. 2022 | 1.31 ± 0.45 | -0.15 | 1.07 ± 0.15 | 0.03 | 0.18 | nr |
| Sittiwicheanwong et al. 2007 | 1.49 ± 0.30 | 0.05 | 1.43 ± 0.28 | 0.07 | 0.02 | p |
| Soori et al. 2017 | 1.32 ± 0.18 | -0.01 | 1.32 ± 0.22 | 0.20 | 0.21 | s |
| Stein et al. 1990 1 | 1.14 ± 0.30 | 0.01 | 1.22 ± 0.31 | -0.03 | -0.04 | p |
| Stein et al. 1990 2 | 1.14 ± 0.30 | 0.01 | 0.93 ± 0.19 | 0.18 | 0.17 | p |
| Suter et al. 1994 | 1.21 ± 0.33 | -0.04 | 1.29 ± 0.35 | 0.01 | 0.05 | s |
| Tan et al. 2016 | 1.35 ± 0.19 | 0.01 | 1.36 ± 0.28 | 0.04 | 0.03 | nr |
| Thompson et al. 2009 | 1.26 ± 0.24 | -0.01 | 1.23 ± 0.20 | 0.06 | 0.07 | p |
| Tsai et al. 2002a | 1.25 ± 0.24 | -0.19 | 1.37 ± 0.48 | 0.17 | 0.36 | nr |
| Tsai et al. 2002b | 1.24 ± 0.26 | -0.05 | 1.38 ± 0.52 | 0.16 | 0.20 | p |
| Tseng et al. 2013 | 1.17 ± 0.13 | -0.01 | 1.25 ± 0.17 | 0.12 | 0.13 | s |
| **Pooled mean values** | **1.26 ± 0.27** | **-0.00 ± 0.22** | **1.25 ± 0.30** | **0.09 ± 0.27** | **0.09 ± 0.10** | **p: 7**  **s: 8**  **nr: 6** |

*?* = Not totally unequivocally reported, LIT = Low-intensity training

**Supplemental table 9.** Absolute pre-values and changes in LDL cholesterol (all changed to mmol/l).

| Study | Pre-  control | Change control | Pre-  LIT | Change  LIT | Change  LIT vs.  control | Plasma (p) Serum (s)  Not reported (nr) |
| --- | --- | --- | --- | --- | --- | --- |
| Eguchi et al. 2012 | 3.38 ± 0.49 | -0.05 | 3.33 ± 0.86 | -0.13 | -0.07 | nr |
| Goncalves et al. 2022 | 2.98 ± 0.26 | -0.18 | 3.57 ± 0.34 | -0.25 | -0.07 | s |
| Huttunen et al. 1979 | 4.83 ± 0.96 | -0.05 | 4.75 ± 1.18 | -0.26 | -0.21 | s |
| Lee et al. 2012 | 2.81 ± 0.70 | -0.09 | 2.69 ± 0.65 | -0.03 | 0.06 | s |
| Lemura et al. 2000 | 3.20 ± 0.20 | -0.10 | 2.60 ± 0.10 | -0.20 | -0.10 | p? |
| Mousavi et al. 2023 | 2.08 ± 0.25 | -0.00 | 2.08 ± 0.38 | -0.25 | -0.25 | s |
| Murphy et al. 2006 | 4.10 ± 1.20 | 0.10 | 4.10 ± 1.10 | -0.10 | -0.20 | s |
| Poon et al. 2022 | 3.47 ± 0.85 | 0.04 | 3.89 ± 0.56 | -0.17 | -0.21 | nr |
| Shaw et al. 2009b | 4.12 ± 0.27 | 0.09 | 3.64 ± 0.92 | -0.77 | -0.86 | nr |
| Sittiwicheanwong et al. 2007 | 3.70 ± 0.81 | -0.17 | 3.65 ± 0.54 | -0.13 | 0.04 | p |
| Soori et al. 2017 | 3.75 ± 0.70 | 0.57 | 4.37 ± 0.57 | -0.78 | -1.34 | s |
| Stein et al. 1990 1 | 3.30 ± 1.11 | 0.21 | 3.73 ± 1.48 | -0.05 | -0.26 | p |
| Stein et al. 1990 2 | 3.30 ± 1.11 | 0.21 | 4.13 ± 0.94 | -0.44 | -0.65 | p |
| Tsai et al. 2002a | 3.36 ± 0.88 | 0.12 | 3.33 ± 0.63 | -0.47 | -0.59 | nr |
| Tsai et al. 2002b | 3.38 ± 0.64 | -0.03 | 3.25 ± 0.72 | -0.46 | -0.42 | p |
| **Pooled mean values** | **3.75 ± 0.79** | **0.01 ± 0.63** | **3.76 ± 0.89** | **-0.29 ± 0.66** | **-0.32 ± 0.31** | **p: 5**  **s: 6**  **nr: 4** |

*? =* Not totally unequivocally reported, LIT = Low-intensity training

**Supplemental table 10.** Absolute pre-values and changes in glucose (all changed to mmol/l).

| Study | Pre-  control | Change control | Pre-  LIT | Change  LIT | Change  LIT vs. control | Plasma (p) Serum (s)  Not reported (nr) |
| --- | --- | --- | --- | --- | --- | --- |
| Blond et al. 2019 | 5.30 ± 0.40 | 0.10 | 5.20 ± 0.40 | 0.10 | 0.00 | p |
| Eguchi et al. 2012 | 5.53 ± 0.57 | -0.11 | 5.38 ± 0.46 | 0.04 | 0.14 | nr |
| Goncalves et al. 2022 | 5.32 ± 0.17 | 0.12 | 5.32 ± 0.11 | -0.01 | -0.13 | p |
| Grieco et al. 2013 | 4.90 ± 0.30 | -0.20 | 4.80 ± 0.50 | 0.00 | 0.20 | p |
| Poon et al. 2022 | 5.48 ± 0.62 | -0.09 | 5.32 ± 0.25 | 0.13 | 0.22 | nr |
| Tan et al. 2016 | 4.94 ± 0.36 | -0.01 | 5.21 ± 0.38 | -0.75 | -0.74 | nr |
| Thompson et al. 2009 | 5.54 ± 0.44 | 0.06 | 5.73 ± 0.55 | -0.03 | -0.09 | p |
| Tseng et al. 2013 | 4.85 ± 0.14 | -0.02 | 4.77 ± 0.16 | -0.27 | -0.24 | s |
| van Aggel-Leijssen 2001 1 | 5.55 ± 0.68 | 0.09 | 5.23 ± 0.38 | 0.09 | 0.00 | p |
| van Aggel-Leijssen 2001 2 | 5.55 ± 0.68 | 0.09 | 4.85 ± 0.49 | 0.24 | 0.15 | p |
| Werner et al. 2019 | 5.33 ± 0.50 | -0.06 | 5.33 ± 0.33 | -0.22 | -0.17 | nr |
| Zhang et al. 2015 | 4.41 ± 0.50 | -0.54 | 4.62 ± 0.31 | 0.02 | 0.56 | s |
| **Pooled mean values** | **5.24 ± 0.46** | **-0.05 ± 0.43** | **5.21 ± 0.39** | **-0.07 ± 0.44** | **-0.05 ± 0.29** | **p: 6**  **s: 2 nr: 4** |

LIT = Low-intensity training

**Supplemental table 11.** Absolute pre-values and changes in triglycerides (all changed to mmol/l).

| Study | Pre-  control | Change control | Pre-  LIT | Change  LIT | Change  LIT vs.  control | Plasma (p)  Serum (s)  Not reported (nr) |
| --- | --- | --- | --- | --- | --- | --- |
| Baghaiee et al. 2016 | 1.85 ± 0.24 | 0.04 | 1.91 ± 0.33 | -0.20 | -0.24 | nr |
| Goncalves et al. 2022 | 1.23 ± 0.23 | -0.11 | 1.40 ± 0.23 | -0.19 | -0.08 | s |
| Huttunen et al. 1979 | 1.44 ± 0.87 | 0.00 | 1.54 ± 0.66 | -0.20 | -0.20 | s |
| Lee et al. 2012 | 1.59 ± 1.13 | -0.56 | 1.57 ± 1.66 | -0.15 | 0.41 | s |
| Lemura et al. 2000 | 1.20 ± 0.00 | 0.00 | 1.40 ± 0.10 | -0.10 | -0.10 | p? |
| Mousavi et al. 2023 | 1.32 ± 0.08 | -0.00 | 1.32 ± 0.11 | -0.20 | -0.20 | s |
| Murphy et al. 2006 | 1.20 ± 0.60 | 0.10 | 1.40 ± 1.20 | 0.00 | -0.10 | s |
| Poon et al. 2022 | 1.40 ± 0.55 | 0.13 | 1.77 ± 0.57 | -0.22 | -0.35 | nr |
| Sittiwicheanwong et al. 2007 | 1.24 ± 0.31 | 0.08 | 1.32 ± 0.26 | -0.12 | -0.20 | p |
| Soori et al. 2017 | 1.21 ± 0.61 | -0.02 | 1.35 ± 0.23 | -0.20 | -0.18 | s |
| Stein et al. 1990 1 | 1.38 ± 0.75 | 0.05 | 1.31 ± 0.53 | -0.17 | -0.22 | p |
| Stein et al. 1990 2 | 1.38 ± 0.75 | 0.05 | 1.19 ± 0.68 | 0.04 | -0.01 | p |
| Suter et al. 1994 | 1.38 ± 0.87 | 0.06 | 1.43 ± 0.60 | 0.32 | 0.26 | s |
| Tan et al. 2016 | 1.36 ± 0.42 | 0.01 | 1.38 ± 0.30 | -0.32 | -0.33 | nr |
| Thompson et al. 2009 | 1.75 ± 0.81 | -0.31 | 1.62 ± 0.68 | -0.19 | 0.12 | p |
| Tsai et al. 2002a | 1.43 ± 0.47 | 0.02 | 1.51 ± 0.42 | -0.20 | -0.21 | nr |
| Tsai et al. 2002b | 1.49 ± 0.68 | -0.13 | 1.26 ± 0.33 | -0.14 | -0.02 | p |
| Tseng et al. 2013 | 1.13 ± 0.13 | 0.00 | 1.04 ± 0.08 | -0.07 | -0.08 | s |
| Zhang et al. 2015 | 0.79 ± 0.22 | 0.15 | 0.87 ± 0.24 | 0.11 | -0.04 | s |
| **Pooled mean values** | **1.38 ± 0.65** | **-0.02 ± 0.51** | **1.41 ± 0.62** | **-0.10 ± 0.47** | **-0.09 ± 0.18** | **p: 6 s: 9 nr: 4** |

*? =* Not totally unequivocally reported, LIT = Low-intensity training

**Supplemental table 12.** Absolute pre-values and changes in systolic blood pressure (mmHg).

| Study | Pre-  control | Change control | Pre-  LIT | Change  LIT | Change  LIT vs. control |
| --- | --- | --- | --- | --- | --- |
| Eguchi et al. 2012 | 128.1 ± 21.2 | -5.8 | 123.8 ± 21.7 | -10.5 | -4.7 |
| Goncalves et al. 2022 | 122.0 ± 5.0 | -7.0 | 120.6 ± 4.0 | -0.6 | 6.4 |
| Gormley et al. 2008 | 109.0 ± 14.0 | -3.0 | 107.0 ± 13.0 | 1.0 | 4.0 |
| Huang et al. 2019b | 116.0 ± 11.6 | 2.0 | 117.0 ± 11.6 | -4.0 | -6.0 |
| McDonald et al. 1993 | 110.0 ± 2.0 | 3.0 | 117.0 ± 9.0 | -8.0 | -11.0 |
| Murphy et al. 2006 | 116.5 ± 13.0 | 2.0 | 120.4 ± 19.7 | -5.0 | -7.0 |
| Poon et al. 2022 | 127.8 ± 7.0 | -7.9 | 115.3 ± 10.0 | -0.3 | 7.6 |
| Rogers et al. 1996 | 140.0 ± 8.9 | -1.3 | 140.0 ± 9.1 | -14.1 | -12.8 |
| Thompson et al. 2009 | 130.0 ± 11.0 | -4.0 | 131.0 ± 12.0 | -2.0 | 2.0 |
| Tsai et al. 2002a | 144.0 ± 13.0 | -8.0 | 139.1 ± 11.4 | -18.1 | -10.1 |
| Tsai et al. 2002b | 137.6 ± 7.9 | -2.9 | 134.3 ± 12.2 | -10.8 | -8.0 |
| Tseng et al. 2013 | 126.0 ± 4.1 | 0.4 | 126.7 ± 6.6 | -7.5 | -7.9 |
| Wang et al. 1995 | 104.9 ± 10.5 | -3.3 | 107.9 ± 10.4 | -15.2 | -11.9 |
| Wang et al. 1997 | 101.1 ± 2.7 | -1.1 | 101.0 ± 5.5 | -8.1 | -7.0 |
| Wang et al. 2005 | 116.0 ± 15.5 | -1.0 | 115.0 ± 11.6 | -11.0 | -10.0 |
| Werner et al. 2019 | 131.0 ± 16.0 | -1.0 | 132.0 ± 15.0 | -1.0 | 0.0 |
| Wu et al. 2017 | 123.0 ± 7.8 | -1.0 | 121.0 ± 11.6 | -5.0 | -4.0 |
| **Pooled mean values** | **124.1 ± 12.2** | **-2.3 ±12.2** | **122.9 ± 12.8** | **-6.3 ± 9.8** | **-4.0 ± 5.8** |

LIT = Low-intensity training

**Supplemental table 13.** Absolute pre-values and changes in diastolic blood pressure (mmHg).

| Study | Pre-  control | Change control | Pre-  LIT | Change  LIT | Change  LIT vs. control |
| --- | --- | --- | --- | --- | --- |
| Eguchi et al. 2012 | 78.1 ± 12.9 | -4.0 | 78.1 ± 11.2 | -5.0 | -1.0 |
| Goncalves et al. 2022 | 78.6 ± 4.0 | -0.8 | 72.9 ± 3.0 | -0.4 | 0.4 |
| Gormley et al. 2008 | 63.0 ± 5.0 | 0.0 | 64.0 ± 6.0 | 2.0 | 2.0 |
| Huang et al. 2019b | 75.0 ± 11.6 | -1.0 | 76.0 ± 11.6 | -2.0 | -1.0 |
| McDonald et al. 1993 | 58.0 ± 4.0 | 7.0 | 63.0 ± 9.0 | -5.0 | -12.0 |
| Murphy et al. 2006 | 74.6 ± 9.0 | -1.0 | 77.2 ± 9.4 | -1.2 | -0.2 |
| Poon et al. 2022 | 80.9 ± 5.3 | -0.8 | 72.1 ± 6.5 | -0.7 | 0.1 |
| Rogers et al. 1996 | 93.0 ± 6.0 | -3.2 | 93.0 ± 8.1 | -5.8 | -2.6 |
| Thompson et al. 2009 | 84.0 ± 9.0 | 1.0 | 88.0 ± 9.0 | -2.0 | -3.0 |
| Tsai et al. 2002a | 97.1 ± 7.6 | 0.3 | 99.5 ± 8.0 | -10.0 | -10.3 |
| Tsai et al. 2002b | 91.6 ± 7.9 | 4.0 | 85.3 ± 10.2 | -5.3 | -9.3 |
| Tseng et al. 2013 | 81.7 ± 5.1 | -0.2 | 81.9 ± 6.6 | -5.8 | -5.6 |
| Wang et al. 1995 | 60.0 ± 6.9 | 0.5 | 63.0 ± 6.6 | -5.5 | -6.0 |
| Wang et al. 1997 | 67.0 ± 2.8 | -2.1 | 67.0 ± 5.6 | -9.2 | -7.1 |
| Wang et al. 2005 | 74.0 ± 11.6 | 2.0 | 72.0 ± 7.8 | -7.0 | -9.0 |
| Werner et al. 2019 | 84.0 ± 10.0 | -2.0 | 86.0 ± 11.0 | -3.0 | -1.0 |
| Wu et al. 2017 | 75.0 ± 11.6 | -1.0 | 74.0 ± 11.6 | -2.0 | -1.0 |
| **Pooled mean values** | **78.9 ± 8.9** | **-0.1 ± 8.4** | **78.3 ± 9.0** | **-3.6 ± 7.1** | **-3.6 ± 4.0** |

LIT = Low-intensity training

**Supplemental table 14.** VO_2max_(ml/kg/min) subgroup analysis. Effect size represents the effect of the low-intensity training group compared to the control group.

| **Subgroup** | **No. of trials / Total no.** | **Subjects / Total no.** | **Effect size (95% CI)** | **95% PI** | **p-value** | **Interaction Homogeneity Q / p-value** |
| --- | --- | --- | --- | --- | --- | --- |
| *All studies* | 37 | 562 | 0.94 (0.74 to 1.13) | -0.07 to 1.94 | <0.001 |  |
| Sex |  |  |  |  |  |  |
| Female | 11/27 | 159 / 373 | 1.44 (0.88 to 2.00) | -0.58 to 3.46 | <0.001 | Q = 2.3 /  p = 0.13 |
| Male | 16/27 | 214 / 373 | 0.97 (0.72 to 1.21) | 0.16 to 1.78 | <0.001 |  |
| *Training volume / week (h)* |  |  |  |  |  |  |
| $\leq$ 2.5 | 13/37 | 192/562 | 0.95 (0.61 to 1.29) | -0.20 to 2.10 | <0.001 | Q = 3.6 /  p = 0.17 |
| 2.6–4 | 19/37 | 262/562 | 0.98 (0.68 to 1.28) | -0.24 to 2.20 | <0.001 |  |
| > 4 | 5/37 | 108/562 | 0.68 (0.47 to 0.88) | 0.33 to 1.02 | <0.001 |  |
| *Duration of exercise (min)* |  |  |  |  |  |  |
| $\leq$ 30 | 16/37 | 207/562 | 0.91 (0.67 to 1.16) | 0.13 to 1.70 | <0.001 | Q = 0.4 /  p = 0.83 |
| 31–50 | 11/37 | 180/562 | 0.98 (0.57 to 1.39) | -0.43 to 2.40 | <0.001 |  |
| > 50 | 10/37 | 175/562 | 0.94 (0.47 to 1.41) | -0.70 to 2.59 | <0.001 |  |
| *Training frequency (/week)* |  |  |  |  |  |  |
| $\leq$ 3 | 13/36 | 169/563 | 1.14 (0.68 to 1.61) | -0.55 to 2.84 | <0.001 | Q = 3.7 /  p = 0.16 |
| 4–4.9 | 7/36 | 118/563 | 0.93 (0.49 to 1.37) | -0.46 to 2.32 | <0.001 |  |
| $\geq$ 5 | 16/36 | 276/563 | 0.71 (0.47 to 0.95) | 0.57 to 0.85 | <0.001 |  |
| *Exercise intensity (% VO_2max_)* |  |  |  |  |  |  |
| $<$ 50 | 6/35 | 91/544 | 0.59 (0.38 to 0.79) | 0.29 to 0.88 | <0.001 | **Q = 7.7 /**  **p = 0.02** |
| 50–55 | 13/35 | 189/544 | 1.00 (0.55 to 1.44) | -0.66 to 2.65 | <0.001 |  |
| > 55 | 16/35 | 264/544 | 1.03 (0.77 to 1.29) | 0.10 to 1.95 | <0.001 |  |
| *Banister’s TRIMP (a.u.)* |  |  |  |  |  |  |
| $\leq$ 3 | 9/35 | 95/540 | 1.09 (0.52 to 1.67) | -0.82 to 3.01 | 0.002 | **Q = 6.9 /**  **p = 0.03** |
| 3.1–4.5 | 12/35 | 207/540 | 0.64 (0.49 to 0.79) | 0.46 to 0.81 | <0.001 |  |
| *> 4.5* | 14/35 | 238/540 | 1.02 (0.73 to 1.31) | -0.01 to 2.06 | <0.001 |  |
| *Duration of intervention (weeks)* |  |  |  |  |  |  |
| $\leq$ 6 | 7/37 | 90/562 | 0.74 (0.33 to 1.15) | -0.52 to 2.01 | <0.001 | Q = 2.4 /  p = 0.30 |
| 7–11 | 9/37 | 102/562 | 1.23 (0.77 to 1.69) | -0.20 to 2.66 | <0.001 |  |
| $\geq$ 12 | 21/37 | 370/562 | 0.92 (0.65 to 1.18) | -0.19 to 2.02 | <0.001 |  |
| *Age (y)* |  |  |  |  |  |  |
| $\leq$ 45 | 30/37 | 428/562 | 1.04 (0.79 to 1.30) | -0.20 to 2.29 | <0.001 | **Q = 7.0 /**  **p = 0.01** |
| 45.1–64.9 | 7/37 | 134/562 | 0.63 (0.45 to 0.80) | 0.40 to 0.86 | <0.001 |  |
| *Body mass index (kg/m^2^)* |  |  |  |  |  |  |
| $\leq$ 25 | 10/26 | 173/404 | 0.73 (0.49 to 0.97) | 0.08 to 1.39 | <0.001 | Q = 3.0 /  p = 0.09 |
| 25.1–34.9 | 16/26 | 231/404 | 1.12 (0.75 to 1.49) | -0.32 to 2.56 | <0.001 |  |
| *VO_2max_ (ml/kg/min)* |  |  |  |  |  |  |
| $<$ 30 | 14/37 | 230/562 | 1.13 (0.66 to 1.61) | -0.73 to 3.00 | <0.001 | Q = 1.5 /  p = 0.48 |
| 30–40 | 18/37 | 251/562 | 0.83 (0.63 to 1.03) | 0.20 to 1.46 | <0.001 |  |
| > 40 | 5/37 | 81/562 | 1.00 (0.42 to 1.59) | -0.92 to 2.92 | <0.001 |  |

**Supplemental table 15.** VO_2max_ (l/min) subgroup analysis. Effect size represents the effect of the low-intensity training group compared to the control group.

| **Subgroup** | **No. of trials / Total no.** | **Subjects / Total no.** | **Effect size (95% CI)** | **95% PI** | **p- value** | **Interaction Homogeneity Q / p-value** |
| --- | --- | --- | --- | --- | --- | --- |
| *All studies* | 7 | 71 | 0.84 (0.36 to 1.33) | -0.66 to 2.35 | <0.001 |  |
| Sex |  |  |  |  |  |  |
| Female |  |  |  |  |  |  |
| Male |  |  |  |  |  |  |
| *Training volume / week (h)* |  |  |  |  |  |  |
| $\leq$ 2.5 | 2/6 | 27/61 | 0.88 (0.42 to 1.33) |  | <0.001 | Q = 0.4 /  p = 0.53 |
| 2.6–4 | 4/6 | 34/61 | 0.61 (-0.07 to 1.29) | -2.3 to 3.52 | 0.079 |  |
| > 4 |  |  |  |  |  |  |
| *Duration of exercise (min)* |  |  |  |  |  |  |
| $\leq$ 30 | 3/7 | 33/71 | 0.72 (0.31 to 1.14) | -2.27 to 3.72 | <0.001 | Q = 0.3 /  p = 0.57 |
| 31–50 |  |  |  |  |  |  |
| > 50 | 4/7 | 38/71 | 1.00 (0.12 to 1.88) | -2.95 to 4.96 | 0.026 |  |
| *Training frequency (/week)* |  |  |  |  |  |  |
| $\leq$ 3 | 4/6 | 47/63 | 0.79 (0.17 to 1.42) | -1.87 to 3.45 | 0.013 | Q = 0.1 /  p = 0.72 |
| 4–4.9 |  |  |  |  |  |  |
| $\geq$ 5 | 2/6 | 16/63 | 1.13 (-0.6 to 2.85) |  | 0.20 |  |
| *Exercise intensity (% VO_2max_)* |  |  |  |  |  |  |
| $<$ 50 | 2/5 | 13/55 | 0.27 (-0.29 to 0.84) |  | 0.338 | **Q = 4.85 /**  **p = 0.03** |
| 50–55 |  |  |  |  |  |  |
| > 55 | 3/5 | 42/55 | 1.08 (0.63 to 1.53) | -2.89 to 5.05 | <0.001 |  |
| *Banister’s TRIMP (a.u.)* |  |  |  |  |  |  |
| $\leq$ 3 | 3/7 | 32/71 | 0.56 (-0.02 to 1.13) | -5.45 to 6.56 | 0.059 | **Q = 11.1 /**  **p < 0.001** |
| 3.1–4.5 | 2/7 | 14/71 | 0.48 (-0.08 to 1.04) |  | 0.096 |  |
| *> 4.5* | 2/7 | 25/71 | 1.68 (1.12 to 2.23) |  | <0.001 |  |
| *Duration of intervention (weeks)* |  |  |  |  |  |  |
| $\leq$ 6 |  |  |  |  |  |  |
| 7–11 |  |  |  |  |  |  |
| $\geq$ 12 |  |  |  |  |  |  |
| *Age (y)* |  |  |  |  |  |  |
| $\leq$ 45 |  |  |  |  |  |  |
| 45.1–64.9 |  |  |  |  |  |  |
| *Body mass index (kg/m^2^)* |  |  |  |  |  |  |
| $\leq$ 25 |  |  |  |  |  |  |
| 25.1–34.9 |  |  |  |  |  |  |
| *VO_2max_ (ml/kg/min)* |  |  |  |  |  |  |
| $<$ 30 |  |  |  |  |  |  |
| 30–40 |  |  |  |  |  |  |
| > 40 |  |  |  |  |  |  |

**Supplemental table 16.** P_max_(W) subgroup analysis. Effect size represents the effect of the low-intensity training group compared to the control group.

| **Subgroup** | **No. of trials / Total no.** | **Subjects / Total no.** | **Effect size (95% CI)** | **95% PI** | **p-value** | **Interaction Homogeneity Q / p-value** |
| --- | --- | --- | --- | --- | --- | --- |
| *All studies* | 9 | 110 | 1.09 (0.86 to 1.31) | 0.82 to 1.36 | <0.001 |  |
| Sex |  |  |  |  |  |  |
| Female |  |  |  |  |  |  |
| Male |  |  |  |  |  |  |
| *Training volume / week (h)* |  |  |  |  |  |  |
| $\leq$ 2.5 |  |  |  |  |  |  |
| 2.6–4 |  |  |  |  |  |  |
| > 4 |  |  |  |  |  |  |
| *Duration of exercise (min)* |  |  |  |  |  |  |
| $\leq$ 30 |  |  |  |  |  |  |
| 31–50 |  |  |  |  |  |  |
| > 50 |  |  |  |  |  |  |
| *Training frequency (/week)* |  |  |  |  |  |  |
| $\leq$ 3 |  |  |  |  |  |  |
| 4–4.9 |  |  |  |  |  |  |
| $\geq$ 5 |  |  |  |  |  |  |
| *Exercise intensity (% VO_2max_)* |  |  |  |  |  |  |
| $<$ 50 | 3/9 | 28/110 | 1.49 (0.45 to 2.53) | -10.58 to 13.57 | 0.005 | Q = 0.7 /  p = 0.42 |
| 50–55 | 6/9 | 82/110 | 1.05 (0.8 to 1.3) | 0.69 to 1.41 | <0.001 |  |
| > 55 | 3/9 | 28/110 | 1.49 (0.45 to 2.53) | -10.58 to 13.57 | 0.005 |  |
| *Banister’s TRIMP (a.u.)* |  |  |  |  |  |  |
| $\leq$ 3 |  |  |  |  |  |  |
| 3.1–4.5 |  |  |  |  |  |  |
| *> 4.5* |  |  |  |  |  |  |
| *Duration of intervention (weeks)* |  |  |  |  |  |  |
| $\leq$ 6 | 5/8 | 66/100 | 0.92 (0.64 to 1.19) | 0.47 to 1.36 | <0.001 | Q = 2.8 /  p = 0.10 |
| 7–11 | 3/8 | 34/100 | 1.53 (0.86 to 2.21) | -5.36 to 8.42 | <0.001 |  |
| $\geq$ 12 |  |  |  |  |  |  |
| *Age (y)* |  |  |  |  |  |  |
| $\leq$ 45 |  |  |  |  |  |  |
| 45.1–64.9 |  |  |  |  |  |  |
| *Body mass index (kg/m^2^)* |  |  |  |  |  |  |
| $\leq$ 25 |  |  |  |  |  |  |
| 25.1–34.9 |  |  |  |  |  |  |
| *VO_2max_ (ml/kg/min)* |  |  |  |  |  |  |
| $<$ 30 |  |  |  |  |  | Q = 2.8 /  p = 0.09 |
| 30–40 | 2/8 | 18/102 | 0.65 (0.14 to 1.16) |  | 0.013 |  |
| > 40 | 6/8 | 84/102 | 1.14 (0.88 to 1.39) | 0.78 to 1.5 | <0.001 |  |

**Supplemental table 17.** Total cholesterol subgroup analysis. Effect size represents the effect of the low-intensity training group compared to the control group.

| **Subgroup** | **No. of trials / Total no.** | **Subjects / Total no.** | **Effect size (95% CI)** | **95% PI** | **p- value** | **Interaction Homogeneity Q / p-value** |
| --- | --- | --- | --- | --- | --- | --- |
| *All studies* | 20 | 322 | -0.29 (-0.50 to -0.09) | -1.03 to 0.45 | 0.005 |  |
| Sex |  |  |  |  |  |  |
| Female | 7/15 | 81/231 | -0.45 (-0.89 to 0.00) | -1.78 to 0.89 | 0.05 | Q = 1.2 /  p = 0.27 |
| Male | 8/15 | 150/231 | -0.14 (-0.44 to 0.16) | -0.99 to 0.71 | 0.35 |  |
| *Training volume / week (h)* |  |  |  |  |  |  |
| $\leq$ 2.5 | 16/19 | 261/314 | -0.39 (-0.63 to -0.16) | -1.19 to 0.4 | <0.001 | **Q = 3.8 /**  **p = 0.05** |
| 2.6–4 | 3/19 | 53/314 | 0.01 (-0.32 to 0.34) | -2.13 to 2.14 | 0.97 |  |
| > 4 |  |  |  |  |  |  |
| *Duration of exercise (min)* |  |  |  |  |  |  |
| $\leq$ 30 | 10/20 | 181/322 | -0.41 (-0.66 to -0.17) | -1.07 to 0.24 | <0.001 | **Q = 6.6 /**  **p = 0.04** |
| 31–50 | 8/20 | 123/322 | -0.27 (-0.65 to 0.12) | -1.46 to 0.93 | 0.17 |  |
| > 50 | 2/20 | 18/322 | 0.4 (-0.17 to 0.97) |  | 0.17 |  |
| *Training frequency (/week)* |  |  |  |  |  |  |
| $\leq$ 3 | 14/19 | 229/314 | -0.41 (-0.62 to -0.2) | -1.02 to 0.2 | <0.001 | Q = 3.1 /  p = 0.21 |
| 4–4.9 | 3/19 | 42/314 | -0.08 (-0.87 to 0.70) | -9.3 to 9.14 | 0.83 |  |
| $\geq$ 5 | 2/19 | 43/314 | -0.05 (-0.41 to 0.32) |  | 0.79 |  |
| *Exercise intensity (% VO_2max_)* |  |  |  |  |  |  |
| $<$ 50 | 7/18 | 135/301 | -0.44 (-0.79 to -0.09) | -1.47 to 0.59 | 0.01 | Q = 3.8 /  p = 0.15 |
| 50–55 | 7/18 | 96/301 | -0.01 (-0.35 to 0.33) | -0.92 to 0.89 | 0.95 |  |
| > 55 | 4/18 | 70/301 | -0.37 (-0.64 to -0.11) | -0.95 to 0.21 | 0.006 |  |
| *Banister’s TRIMP (a.u.)* |  |  |  |  |  |  |
| $\leq$ 3 | 12/19 | 190/314 | -0.52 (-0.79 to -0.25) | -1.34 to 0.3 | <0.001 | **Q = 6.1 /**  **p = 0.01** |
| 3.1–4.5 | 7/19 | 124/314 | -0.04 (-0.28 to 0.20) | -0.56 to 0.48 | 0.73 |  |
| *> 4.5* |  |  |  |  |  |  |
| *Duration of intervention (weeks)* |  |  |  |  |  |  |
| $\leq$ 6 |  |  |  |  |  | Q = 1.3 /  p = 0.25 |
| 7–11 | 6/19 | 108/312 | -0.6 (-1.19 to -0.02) | -2.56 to 1.35 | 0.04 |  |
| $\geq$ 12 | 13/19 | 204/312 | -0.24 (-0.47 to -0.01) | -0.92 to 0.44 | 0.04 |  |
| *Age (y)* |  |  |  |  |  |  |
| $\leq$ 45 | 13/20 | 213/322 | -0.27 (-0.53 to -0.01) | -1.11 to 0.57 | 0.05 | Q = 0.13 /  p = 0.72 |
| 45.1–64.9 | 7/20 | 109/322 | -0.35 (-0.72 to 0.02) | -1.45 to 0.75 | 0.06 |  |
| *Body mass index (kg/m^2^)* |  |  |  |  |  |  |
| $\leq$ 25 | 6/17 | 116/251 | -0.51 (-0.88 to -0.15) | -1.64 to 0.61 | 0.006 | Q = 1.7 /  p = 0.19 |
| 25.1–34.9 | 11/17 | 135/251 | -0.18 (-0.51 to 0.15) | -1.22 to 0.86 | 0.28 |  |
| *VO_2max_ (ml/kg/min)* |  |  |  |  |  |  |
| $<$ 30 | 2/10 | 21/151 | -1.02 (-2.6 to 0.57) |  | 0.21 | Q = 1.1 /  p = 0.31 |
| 30–40 | 8/10 | 130/151 | -0.18 (-0.45 to 0.10) | -0.9 to 0.54 | 0.20 |  |
| > 40 |  |  |  |  |  |  |

**Supplemental table 18.** HDL subgroup analysis. Effect size represents the effect of the low-intensity training group compared to the control group.

| **Subgroup** | **No. of trials / Total no.** | **Subjects / Total no.** | **Effect size (95% CI)** | **95% PI** | **p-value** | **Interaction Homogeneity Q / p-value** |
| --- | --- | --- | --- | --- | --- | --- |
| *All studies* | 21 | 323 | 0.32 (0.16 to 0.48) | -0.18 to 0.83 | <0.001 |  |
| Sex |  |  |  |  |  |  |
| Female | 6/17 | 69/258 | 0.16 (-0.09 to 0.41) | -0.35 to 1.23 | 0.21 | Q = 2.3 /  p = 0.13 |
| Male | 11/17 | 189/258 | 0.44 (0.18 to 0.70) | 0.15 to 0.44 | <0.001 |  |
| *Training volume / week (h)* |  |  |  |  |  |  |
| $\leq$ 2.5 | 15/21 | 242/323 | 0.28 (0.11 to 0.45) | -0.16 to 0.73 | 0.001 | Q = 1.8 /  p = 0.41 |
| 2.6–4 | 4/21 | 63/323 | 1.06 (-0.76 to 2.88) | -7.65 to 9.77 | 0.25 |  |
| > 4 | 2/21 | 18/323 | 0.58 (0.07 to 1.08) |  | 0.03 |  |
| *Duration of exercise (min)* |  |  |  |  |  |  |
| $\leq$ 30 | 10/21 | 181/323 | 0.27 (0.11 to 0.42) | 0.08 to 0.45 | <0.001 | Q = 1.7 /  p = 0.43 |
| 31–50 | 8/21 | 114/323 | 0.66 (-0.08 to 1.41) | -1.98 to 3.31 | 0.08 |  |
| > 50 | 3/21 | 28/323 | 0.46 (0.05 to 0.87) | -2.2 to 3.12 | 0.03 |  |
| *Training frequency (/week)* |  |  |  |  |  |  |
| $\leq$ 3 | 14/20 | 222/315 | 0.29 (0.09 to 0.48) | -0.25 to 0.82 | 0.003 | Q = 0.9 /  p = 0.63 |
| 4–4.9 | 3/20 | 40/315 | 1.48 (-0.98 to 3.95) | -29.76 to 32.73 | 0.24 |  |
| $\geq$ 5 | 3/20 | 53/315 | 0.27 (-0.06 to 0.61) | -2.38 to 2.93 | 0.11 |  |
| *Exercise intensity (% VO_2max_)* |  |  |  |  |  |  |
| $<$ 50 | 9/19 | 155/302 | 0.64 (0.01 to 1.28) | -1.64 to 2.92 | 0.05 | Q = 1.9 /  p = 0.39 |
| 50–55 | 6/19 | 84/302 | 0.23 (0.00 to 0.46) | -0.09 to 0.55 | 0.05 |  |
| > 55 | 4/19 | 63/302 | 0.16 (-0.10 to 0.43) | -0.42 to 0.74 | 0.23 |  |
| *Banister’s TRIMP (a.u.)* |  |  |  |  |  |  |
| $\leq$ 3 | 12/20 | 197/313 | 0.23 (0.03 to 0.42) | -0.26 to 0.71 | 0.02 | Q = 1.7 /  p = 0.42 |
| 3.1–4.5 | 6/20 | 98/313 | 0.32 (0.10 to 0.54) | 0.01 to 0.63 | 0.005 |  |
| *> 4.5* | 2/20 | 18/313 | 0.58 (0.07 to 1.08) |  | 0.03 |  |
| *Duration of intervention (weeks)* |  |  |  |  |  |  |
| $\leq$ 6 |  |  |  |  |  | Q = 0.5 /  p = 0.47 |
| 7–11 | 7/20 | 118/313 | 0.67 (-0.25 to 1.59) | -2.6 to 3.94 | 0.16 |  |
| $\geq$ 12 | 13/20 | 195/313 | 0.32 (0.16 to 0.49) | 0.01 to 0.63 | <0.001 |  |
| *Age (y)* |  |  |  |  |  |  |
| $\leq$ 45 | 14/21 | 230/323 | 0.26 (0.11 to 0.42) | -0.05 to 0.57 | 0.001 | Q = 1.3 /  p = 0.26 |
| 45.1–64.9 | 7/21 | 93/323 | 0.78 (-0.11 to 1.66) | -2.34 to 3.89 | 0.09 |  |
| *Body mass index (kg/m^2^)* |  |  |  |  |  |  |
| $\leq$ 25 | 6/18 | 109/252 | 0.2 (-0.01 to 0.4) | -0.09 to 0.48 | 0.06 | Q = 2.4 /  p = 0.12 |
| 25.1–34.9 | 12/18 | 143/252 | 0.54 (0.15 to 0.94) | -0.85 to 1.94 | 0.007 |  |
| *VO_2max_ (ml/kg/min)* |  |  |  |  |  |  |
| $<$ 30 | 3/9 | 31/123 | 1.59 (-0.89 to 4.06) | -31.76 to 34.5 | 0.21 | Q = 1.1 /  p = 0.30 |
| 30–40 | 6/9 | 92/123 | 0.29 (0.07 to 0.51) | -0.03 to 0.6 | 0.01 |  |
| > 40 |  |  |  |  |  |  |

| **Subgroup** | **No. of trials / Total no.** | **Subjects / Total no.** | **Effect size (95% CI)** | **95% PI** | **p- value** | **Interaction Homogeneity Q / p-value** |
| --- | --- | --- | --- | --- | --- | --- |
| *All studies* | 15 | 223 | -0.42 (-0.62 to -0.22) | -1.00 to 0.16 | <0.001 |  |
| Sex |  |  |  |  |  |  |
| Female | 5/12 | 54/168 | -0.42 (-1.06 to 0.22) | -2.7 to 1.86 | 0.20 | Q = 0 /  p = 0.97 |
| Male | 7/12 | 114/168 | -0.43 (-0.70 to -0.16) | -1.09 to 0.23 | 0.002 |  |
| *Training volume / week (h)* |  |  |  |  |  |  |
| $\leq$ 2.5 |  |  |  |  |  |  |
| 2.6–4 |  |  |  |  |  |  |
| > 4 |  |  |  |  |  |  |
| *Duration of exercise (min)* |  |  |  |  |  |  |
| $\leq$ 30 | 9/14 | 153/215 | -0.37 (-0.57 to -0.17) | -0.79 to 0.06 | <0.001 | Q = 0.9 /  p = 0.34 |
| 31–50 | 5/14 | 62/215 | -0.69 (-1.32 to -0.06) | -2.91 to 1.53 | 0.03 |  |
| > 50 |  |  |  |  |  |  |
| *Training frequency (/week)* |  |  |  |  |  |  |
| $\leq$ 3 |  |  |  |  |  |  |
| 4–4.9 |  |  |  |  |  |  |
| $\geq$ 5 |  |  |  |  |  |  |
| *Exercise intensity (% VO_2max_)* |  |  |  |  |  |  |
| $<$ 50 | 6/12 | 120/190 | -0.50 (-0.85 to -0.14) | -1.57 to 0.58 | 0.006 | Q = 2.0 /  p = 0.37 |
| 50–55 | 3/12 | 26/190 | -0.12 (-0.51 to 0.28) | -2.65 to 2.42 | 0.56 |  |
| > 55 | 3/12 | 44/190 | -0.35 (-0.81 to 0.12) | -5.13 to 4.43 | 0.15 |  |
| *Banister’s TRIMP (a.u.)* |  |  |  |  |  |  |
| $\leq$ 3 | 11/13 | 178/203 | -0.38 (-0.59 to -0.17) | -0.89 to 0.13 | <0.001 | Q = 0.0 /  p = 0.84 |
| 3.1–4.5 | 2/13 | 25/203 | -0.43 (-0.88 to 0.02) |  | 0.06 |  |
| *> 4.5* |  |  |  |  |  |  |
| *Duration of intervention (weeks)* |  |  |  |  |  |  |
| $\leq$ 6 |  |  |  |  |  | Q = 0.6 /  p = 0.46 |
| 7–11 | 5/15 | 93/223 | -0.59 (-1.09 to -0.09) | -2.29 to 1.11 | 0.02 |  |
| $\geq$ 12 | 10/15 | 130/223 | -0.38 (-0.62 to -0.14) | -1.01 to 0.25 | 0.002 |  |
| *Age (y)* |  |  |  |  |  |  |
| $\leq$ 45 | 11/15 | 175/223 | -0.4 (-0.58 to -0.21) | -0.79 to -0.01 | <0.001 | Q = 0.3 /  p = 0.62 |
| 45.1–64.9 | 4/15 | 48/223 | -0.61 (-1.44 to 0.21) | -4.33 to 3.11 | 0.15 |  |
| *Body mass index (kg/m^2^)* |  |  |  |  |  |  |
| $\leq$ 25 | 4/11 | 62/140 | -0.44 (-0.81 to -0.06) | -1.83 to 0.96 | 0.02 | Q = 0.0 /  p = 0.85 |
| 25.1–34.9 | 7/11 | 78/140 | -0.39 (-0.74 to -0.03) | -1.36 to 0.59 | 0.03 |  |
| *VO_2max_ (ml/kg/min)* |  |  |  |  |  |  |
| $<$ 30 | 3/6 | 33/67 | -0.44 (-1.21 to 0.33) | -9.41 to 8.53 | 0.26 | Q = 0.0 /  p = 0.89 |
| 30–40 | 3/6 | 34/67 | -0.5 (-0.86 to -0.14) | -2.85 to 1.85 | 0.007 |  |
| > 40 |  |  |  |  |  |  |

**Supplemental table 19.** LDL subgroup analysis. Effect size represents the effect of the low-intensity training group compared to the control group.

| **Subgroup** | **No. of trials / Total no.** | **Subjects / Total no.** | **Effect size (95% CI)** | **95% PI** | **p- value** | **Interaction Homogeneity Q / p-value** |
| --- | --- | --- | --- | --- | --- | --- |
| *All studies* | 12 | 174 | -0.18 (-0.66 to 0.30) | -1.91 to 1.55 | 0.47 |  |
| Sex |  |  |  |  |  |  |
| Female | 3/9 | 35/99 | -0.11 (-1.98 to 1.76) | -23.59 to 23.36 | 0.91 | Q = 0.38 /  p = 0.85 |
| Male | 6/9 | 64/99 | -0.31 (-0.88 to 0.27) | -2.03 to 1.42 | 0.29 |  |
| *Training volume / week (h)* |  |  |  |  |  |  |
| $\leq$ 2.5 | 6/12 | 87/174 | 0.07 (-0.50 to 0.64) | -1.76 to 1.89 | 0.82 | Q = 1.1 /  p = 0.57 |
| 2.6–4 | 4/12 | 38/174 | -0.32 (-1.40 to 0.76) | -5.17 to 4.53 | 0.56 |  |
| > 4 | 2/12 | 49/174 | -0.72 (-2.25 to 0.81) |  | 0.36 |  |
| *Duration of exercise (min)* |  |  |  |  |  |  |
| $\leq$ 30 |  |  |  |  |  | Q = 0.36 /  p = 0.85 |
| 31–50 | 6/11 | 94/166 | -0.26 (-1.11 to 0.59) | -3.24 to 2.72 | 0.55 |  |
| > 50 | 5/11 | 72/166 | -0.16 (-0.80 to 0.48) | -2.26 to 1.94 | 0.63 |  |
| *Training frequency (/week)* |  |  |  |  |  |  |
| $\leq$ 3 | 6/12 | 68/174 | -0.11 (-0.49 to 0.27) | -0.96 to 0.74 | 0.57 | Q = 4.3 /  p = 0.12 |
| 4–4.9 | 3/12 | 42/174 | 0.48 (-0.40 to 1.35) | -9.6 to 10.55 | 0.29 |  |
| $\geq$ 5 | 3/12 | 64/174 | -1.11 (-2.32 to 0.11) | -15.89 to 13.68 | 0.08 |  |
| *Exercise intensity (% VO_2max_)* |  |  |  |  |  |  |
| $<$ 50 | 4/10 | 38/137 | -0.82 (-1.89 to 0.24) | -5.6 to 3.95 | 0.131 | Q = 3.7 /  p = 0.055 |
| 50–55 | 6/10 | 99/137 | 0.3 (-0.11 to 0.71) | -0.8 to 1.39 | 0.155 |  |
| > 55 |  |  |  |  |  |  |
| *Banister’s TRIMP (a.u.)* |  |  |  |  |  |  |
| $\leq$ 3 | 5/12 | 43/174 | 0.48 (0.02 to 0.93) | -0.53 to 1.48 | 0.04 | **Q = 7.0 /**  **p = 0.03** |
| 3.1–4.5 | 5/12 | 82/174 | -0.56 (-1.28 to 0.16) | -3.1 to 1.99 | 0.13 |  |
| *> 4.5* | 2/12 | 49/174 | -0.72 (-2.25 to 0.81) |  | 0.36 |  |
| *Duration of intervention (weeks)* |  |  |  |  |  |  |
| $\leq$ 6 |  |  |  |  |  |  |
| 7–11 |  |  |  |  |  |  |
| $\geq$ 12 |  |  |  |  |  |  |
| *Age (y)* |  |  |  |  |  |  |
| $\leq$ 45 | 8/12 | 105/174 | 0.01 (-0.58 to 0.6) | -1.89 to 1.91 | 0.97 | Q = 1.0 /  p = 0.31 |
| 45.1–64.9 | 4/12 | 69/174 | -0.52 (-1.38 to 0.33) | -4.38 to 3.33 | 0.23 |  |
| *Body mass index (kg/m^2^)* |  |  |  |  |  |  |
| $\leq$ 25 |  |  |  |  |  |  |
| 25.1–34.9 |  |  |  |  |  |  |
| *VO_2max_ (ml/kg/min)* |  |  |  |  |  |  |
| $<$ 30 |  |  |  |  |  |  |
| 30–40 |  |  |  |  |  |  |
| > 40 |  |  |  |  |  |  |

**Supplemental table 20.** Glucose subgroup analysis. Effect size represents the effect of the low-intensity training group compared to the control group.

| **Subgroup** | **No. of trials / Total no.** | **Subjects / Total no.** | **Effect size (95% CI)** | **95% PI** | **p- value** | **Interaction Homogeneity Q / p-value** |
| --- | --- | --- | --- | --- | --- | --- |
| *All studies* | 19 | 298 | -0.38 (-0.60 to -0.16) | -1.22 to 0.47 | <0.001 |  |
| Sex |  |  |  |  |  |  |
| Female | 6/16 | 73/243 | -0.55 (-0.99 to -0.11) | -1.92 to 0.82 | 0.01 | Q = 0.5 / p = 0.49 |
| Male | 10/16 | 170/243 | -0.36 (-0.69 to -0.03) | -1.45 to 0.74 | 0.04 |  |
| *Training volume / week (h)* |  |  |  |  |  |  |
| $\leq$ 2.5 | 14/19 | 227/298 | -0.38 (-0.61 to -0.15) | -1.14 to 0.37 | 0.001 | Q = 0.1 / p = 0.94 |
| 2.6–4 | 3/19 | 53/298 | -0.42 (-1.24 to 0.41) | -10.42 to 9.59 | 0.32 |  |
| > 4 | 2/19 | 18/298 | -0.22 (-1.14 to 0.70) |  | 0.65 |  |
| *Duration of exercise (min)* |  |  |  |  |  |  |
| $\leq$ 30 | 9/19 | 173/298 | -0.46 (-0.87 to -0.04) | -1.87 to 0.96 | 0.03 | Q = 0.3 / p = 0.86 |
| 31–50 | 8/19 | 107/298 | -0.35 (-0.62 to -0.07) | -1.06 to 0.37 | 0.01 |  |
| > 50 | 2/19 | 18/298 | -0.22 (-1.14 to 0.70) |  | 0.65 |  |
| *Training frequency (/week)* |  |  |  |  |  |  |
| $\leq$ 3 | 12/18 | 195/290 | -0.46 (-0.71 to -0.21) | -1.23 to 0.32 | <0.001 | Q = 0.6 / p = 0.74 |
| 4–4.9 | 3/18 | 42/290 | -0.22 (-0.76 to 0.32) | -6.16 to 5.72 | 0.42 |  |
| $\geq$ 5 | 3/18 | 53/290 | -0.37 (-1.16 to 0.42) | -9.9 to 9.15 | 0.35 |  |
| *Exercise intensity (% VO_2max_)* |  |  |  |  |  |  |
| $<$ 50 | 9/17 | 155/277 | -0.35 (-0.54 to -0.17) | -0.68 to -0.03 | <0.001 | Q = 5.8 / p = 0.06 |
| 50–55 | 5/17 | 78/277 | 0.05 (-0.27 to 0.36) | -0.83 to 0.92 | 0.78 |  |
| > 55 | 3/17 | 44/277 | -0.69 (-1.46 to 0.09) | -9.84 to 8.47 | 0.08 |  |
| *Banister’s TRIMP (a.u.)* |  |  |  |  |  |  |
| $\leq$ 3 | 11/18 | 182/288 | -0.48 (-0.75 to -0.21) | -1.32 to 0.35 | <0.001 | Q = 2.2 / p = 0.33 |
| 3.1–4.5 | 5/18 | 88/288 | -0.11 (-0.54 to 0.32) | -1.57 to 1.34 | 0.61 |  |
| *> 4.5* | 2/18 | 18/288 | -0.22 (-1.14 to 0.70) |  | 0.65 |  |
| *Duration of intervention (weeks)* |  |  |  |  |  |  |
| $\leq$ 6 |  |  |  |  |  |  |
| 7–11 | 7/19 | 118/298 | -0.74 (-1.2 to -0.29) | -2.19 to 0.71 | 0.001 | **Q = 4.8 / p = 0.03** |
| $\geq$ 12 | 12/19 | 180/298 | -0.18 (-0.40 to 0.03) | -0.79 to 0.43 | 0.10 |  |
| *Age (y)* |  |  |  |  |  |  |
| $\leq$ 45 | 13/19 | 213/298 | -0.33 (-0.62 to -0.04) | -1.34 to 0.68 | 0.02 | Q = 0.4 / p = 0.52 |
| 45.1–64.9 | 6/19 | 85/298 | -0.48 (-0.84 to -0.13) | -1.5 to 0.54 | 0.007 |  |
| *Body mass index (kg/m^2^)* |  |  |  |  |  |  |
| $\leq$ 25 | 5/16 | 90/227 | -0.68 (-1.48 to 0.13) | -3.7 to 2.35 | 0.10 | Q = 0.6 / p = 0.42 |
| 25.1–34.9 | 11/16 | 137/227 | -0.34 (-0.57 to -0.11) | -0.93 to 0.25 | 0.004 |  |
| *VO_2max_ (ml/kg/min)* |  |  |  |  |  |  |
| $<$ 30 | 3/9 | 31/125 | -0.49 (-0.88 to -0.11) | -6.35 to 5.74 | 0.01 | Q = 0.7 / p = 0.41 |
| 30–40 | 6/9 | 94/125 | -0.23 (-0.71 to 0.25) | -1.85 to 1.39 | 0.35 |  |
| > 40 |  |  |  |  |  |  |

**Supplemental table 21.** Triglycerides subgroup analysis. Effect size represents the effect of the low-intensity training group compared to the control group.

| **Subgroup** | **No. of trials / Total no.** | **Subjects / Total no.** | **Effect size (95% CI)** | **95% PI** | **p-value** | **Interaction Homogeneity Q / p-value** |
| --- | --- | --- | --- | --- | --- | --- |
| *All studies* | 17 | 233 | -0.41 (-0.76 to -0.06) | -1.83 to 1.01 | 0.02 |  |
| Sex |  |  |  |  |  |  |
| Female | 2/11 | 16/132 | -0.83 (-2.11 to 0.47) |  | 0.21 | Q = 0.5 /  p = 0.49 |
| Male | 9/11 | 116/132 | -0.32 (-0.92 to 0.28) | -2.41 to 1.77 | 0.29 |  |
| *Training volume / week (h)* |  |  |  |  |  |  |
| $\leq$ 2.5 | 9/16 | 136/223 | -0.11 (-0.59 to 0.38) | -1.77 to 1.56 | 0.67 | Q = 2.7 /  p = 0.10 |
| 2.6–4 | 7/16 | 87/223 | -0.66 (-1.10 to -0.22) | -1.98 to 0.66 | 0.003 |  |
| > 4 |  |  |  |  |  |  |
| *Duration of exercise (min)* |  |  |  |  |  |  |
| $\leq$ 30 | 8/17 | 106/233 | -0.68 (-0.91 to -0.45) | -0.96 to -0.4 | <0.001 | Q = 5.8 /  p = 0.06 |
| 31–50 | 6/17 | 94/233 | 0.15 (-0.49 to 0.79) | -2.04 to 2.33 | 0.65 |  |
| > 50 | 3/17 | 33/233 | -0.74 (-1.87 to 0.39) | -14.23 to 12.75 | 0.20 |  |
| *Training frequency (/week)* |  |  |  |  |  |  |
| $\leq$ 3 | 9/17 | 125/233 | -0.25 (-0.80 to 0.30) | -2.15 to 1.65 | 0.38 | **Q = 16.4 /**  **p < 0.001** |
| 4–4.9 | 2/17 | 34/233 | 0.22 (-0.16 to 0.59) |  | 0.26 |  |
| $\geq$ 5 | 6/17 | 74/233 | -0.82 (-1.16 to -0.48) | -1.62 to -0.02 | <0.001 |  |
| *Exercise intensity (% VO_2max_)* |  |  |  |  |  |  |
| $<$ 50 | 5/16 | 71/222 | -0.78 (-1.09 to -0.47) | -1.36 to -0.2 | <0.001 | Q = 3.9 /  p = 0.14 |
| 50–55 | 5/16 | 60/222 | -0.03 (-0.72 to 0.66) | -2.53 to 2.47 | 0.93 |  |
| > 55 | 6/16 | 91/222 | -0.55 (-0.92 to -0.18) | -1.6 to 0.5 | 0.003 |  |
| *Banister’s TRIMP (a.u.)* |  |  |  |  |  |  |
| $\leq$ 3 | 7/17 | 87/233 | -0.54 (-1.09 to 0.01) | -2.29 to 1.22 | 0.05 | **Q = 14.8 /**  **p < 0.001** |
| 3.1–4.5 | 4/17 | 71/233 | 0.35 (-0.13 to 0.84) | -1.67 to 2.37 | 0.15 |  |
| *> 4.5* | 6/17 | 75/233 | -0.77 (-1.08 to -0.46) | -1.38 to -0.16 | <0.001 |  |
| *Duration of intervention (weeks)* |  |  |  |  |  |  |
| $\leq$ 6 | 3/17 | 44/233 | -0.21 (-0.69 to 0.27) | -5.13 to 4.71 | 0.39 | Q = 5.7 /  p = 0.06 |
| 7–11 | 5/17 | 64/233 | -0.87 (-1.28 to -0.47) | -1.92 to 0.18 | <0.001 |  |
| $\geq$ 12 | 9/17 | 125/233 | -0.21 (-0.78 to 0.37) | -2.2 to 1.79 | 0.48 |  |
| *Age (y)* |  |  |  |  |  |  |
| $\leq$ 45 | 13/17 | 167/233 | -0.49 (-0.95 to -0.04) | -2.22 to 1.23 | 0.04 | Q = 1.4 /  p = 0.24 |
| 45.1–64.9 | 4/17 | 66/233 | -0.14 (-0.51 to 0.23) | -1.51 to 1.23 | 0.46 |  |
| *Body mass index (kg/m^2^)* |  |  |  |  |  |  |
| $\leq$ 25 | 6/14 | 107/208 | -0.34 (-0.67 to -0.01) | -1.3 to 0.63 | 0.04 | Q = 0.2 /  p = 0.69 |
| 25.1–34.9 | 8/14 | 101/208 | -0.19 (-0.84 to 0.46) | -2.41 to 2.03 | 0.56 |  |
| *VO_2max_ (ml/kg/min)* |  |  |  |  |  |  |
| $<$ 30 | 2/10 | 14/140 | -1.43 (-2.19 to -0.67) |  | <0.001 | **Q = 8.4 /**  **p < 0.001** |
| 30–40 | 8/10 | 126/140 | -0.17 (-0.56 to 0.22) | -1.44 to 1.1 | 0.39 |  |
| > 40 |  |  |  |  |  |  |

**Supplemental table 22.** Systolic blood pressure subgroup analysis. Effect size represents the effect of the low-intensity training group compared to the control group.

| **Subgroup** | **No. of trials / Total no.** | **Subjects / Total no.** | **Effect size (95% CI)** | **95% PI** | **p- value** | **Interaction Homogeneity Q / p-value** |
| --- | --- | --- | --- | --- | --- | --- |
| *All studies* | 17 | 233 | -0.43 (-0.68 to -0.18) | -1.38 to 0.48 | <0.001 |  |
| Sex |  |  |  |  |  |  |
| Female | 2/11 | 16/132 | -0.76 (-2.16 to 0.65) |  | 0.29 | Q = 0.2 /  p = 0.64 |
| Male | 9/11 | 116/132 | -0.42 (-0.70 to -0.13) | -1.17 to 0.34 | 0.005 |  |
| *Training volume / week (h)* |  |  |  |  |  |  |
| $\leq$ 2.5 | 9/16 | 136/223 | -0.33 (-0.63 to -0.02) | -1.20 to 0.55 | 0.04 | Q = 0.6 /  p = 0.46 |
| 2.6–4 | 7/16 | 87/223 | -0.55 (-1.05 to -0.05) | -2.14 to 1.05 | 0.03 |  |
| > 4 |  |  |  |  |  |  |
| *Duration of exercise (min)* |  |  |  |  |  |  |
| $\leq$ 30 | 8/17 | 106/233 | -0.67 (-1.05 to -0.3) | -1.80 to 0.46 | <0.001 | **Q = 6.6 /**  **p = 0.04** |
| 31–50 | 6/17 | 94/233 | -0.11 (-0.34 to 0.12) | -0.44 to 0.21 | 0.33 |  |
| > 50 | 3/17 | 33/233 | -0.58 (-1.62 to 0.46) | -12.92 to 11.76 | 0.28 |  |
| *Training frequency (/week)* |  |  |  |  |  |  |
| $\leq$ 3 | 9/17 | 125/233 | -0.40 (-0.76 to -0.04) | -1.49 to 0.70 | 0.03 | Q = 2.5 /  p = 0.28 |
| 4–4.9 | 2/17 | 34/233 | -0.01 (-0.67 to 0.65) |  | 0.97 |  |
| $\geq$ 5 | 6/17 | 74/233 | -0.64 (-1.06 to -0.22) | -1.89 to 0.60 | 0.003 |  |
| *Exercise intensity (% VO_2max_)* |  |  |  |  |  |  |
| $<$ 50 | 5/16 | 71/222 | -0.70 (-1.18 to -0.23) | -2.22 to 0.82 | 0.004 | Q = 1.7 /  p = 0.42 |
| 50–55 | 5/16 | 60/222 | -0.24 (-0.75 to 0.28) | -1.95 to 1.48 | 0.37 |  |
| > 55 | 6/16 | 91/222 | -0.45 (-0.83 to -0.07) | -1.56 to 0.67 | 0.02 |  |
| *Banister’s TRIMP (a.u.)* |  |  |  |  |  |  |
| $\leq$ 3 | 7/17 | 87/233 | -0.57 (-1.03 to -0.10) | -2.00 to 0.86 | 0.02 | **Q = 7.1 /**  **p = 0.03** |
| 3.1–4.5 | 4/17 | 71/233 | -0.05 (-0.31 to 0.20) | -0.61 to 0.51 | 0.68 |  |
| *> 4.5* | 6/17 | 75/233 | -0.61 (-1.00 to -0.21) | -1.73 to 0.52 | 0.003 |  |
| *Duration of intervention (weeks)* |  |  |  |  |  |  |
| $\leq$ 6 | 3/17 | 44/233 | 0.05 (-0.28 to 0.38) | -2.01 to 2.19 | 0.78 | Q = 8.5 /  p = 0.59 |
| 7–11 | 5/17 | 64/233 | -0.83 (-1.37 to -0.29) | -2.56 to 0.90 | 0.002 |  |
| $\geq$ 12 | 9/17 | 125/233 | -0.42 (-0.74 to -0.1) | -1.36 to 0.52 | 0.01 |  |
| *Age (y)* |  |  |  |  |  |  |
| $\leq$ 45 | 13/17 | 167/233 | -0.45 (-0.76 to -0.14) | -1.49 to 0.59 | 0.005 | Q = 0.0 /  p = 0.84 |
| 45.1–64.9 | 4/17 | 66/233 | -0.39 (-0.86 to 0.09) | -2.33 to 1.55 | 0.11 |  |
| *Body mass index (kg/m^2^)* |  |  |  |  |  |  |
| $\leq$ 25 | 6/14 | 107/208 | -0.30 (-0.71 to 0.11) | -1.63 to 1.04 | 0.16 | Q = 0.2 /  p = 0.69 |
| 25.1–34.9 | 8/14 | 101/208 | -0.41 (-0.78 to -0.04) | -1.48 to 0.66 | 0.03 |  |
| *VO_2max_ (ml/kg/min)* |  |  |  |  |  |  |
| $<$ 30 | 2/10 | 14/140 | -0.92 (-2.08 to 0.25) |  | 0.12 | Q = 1.3 /  p = 0.26 |
| 30–40 | 8/10 | 126/140 | -0.23 (-0.49 to 0.04) | -0.92 to 0.47 | 0.09 |  |
| > 40 |  |  |  |  |  |  |

**Supplemental table 23.** Diastolic blood pressure subgroup analysis. Effect size represents the effect of the low-intensity training group compared to the control group.

**Supplemental table 24.** Results of leave-one-out analysis including the minimum and maximum ES and 95% CIs for each outcome.

| Variable | Actual ES (95% CI) | Min – Max ES in LOO | Min – Max 95% CI in LOO |
| --- | --- | --- | --- |
| VO_2max_ (ml/kg/min) | 0.94 (0.74 to 1.13) | 0.88 – 0.96 | 0.71 to 1.16 |
| VO_2max_ (l/min) | 0.84 (0.36 to 1.33) | 0.70 – 0.98 | 0.22 to 1.47 |
| P_max_ | 1.09 (0.86 to 1.31) | 1.04 – 1.14 | 0.81 to 1.38 |
| VT1 | 0.74 (0.26 to 1.22) | 0.63 – 0.91 | 0.09 to 1.48 |
| Total cholesterol | -0.29 (-0.50 to -0.09) | -0.33 – -0.24 | -0.53 to -0.05 |
| HDL | 0.32 (0.16 to 0.48) | 0.27 – 0.35 | 0.14 to 0.52 |
| LDL | -0.42 (-0.62 to -0.22) | -0.46 – -0.35 | -0.68 to -0.18 |
| Glucose | -0.18 (-0.66 to 0.30) | -0.31 – -0.03 | -0.75 to 0.39 |
| Triglycerides | -0.38 (-0.60 to -0.16) | -0.42 – -0.31 | -0.63 to -0.12 |
| Systolic blood pressure | -0.41 (-0.76 to -0.06) | -0.49 – -0.35 | -0.82 to 0.00 |
| Diastolic blood pressure | -0.43 (-0.68 to -0.18) | -0.48 – -0.38 | -0.73 to -0.13 |

VO_2max_ Maximum oxygen uptake; P_max_ peak power of the cycle ergometer test; HDL High-density lipoprotein cholesterol; LDL Low-density lipoprotein cholesterol.

**Supplemental table 25.** Results of leave one out analysis and 95% prediction interval (PI), including the minimum and maximum of 95% PIs for each outcome.

| Variable | Actual ES (95% PI) | Min – Max ES in LOO | Min – Max 95% PI in LOO |
| --- | --- | --- | --- |
| VO_2max_ (ml/kg/min) | 0.94 (-0.07 to 1.94) | 0.88 – 0.96 | -0.11 to 2.00 |
| VO_2max_ (l/min) | 0.84 (-0.67 to 2.34) | 0.70 – 0.98 | -1.11 to 2.76 |
| P_max_ | 1.09 (0.82 to 1.36) | 1.04 – 1.14 | 0.75 to 1.44 |
| VT1 | 0.74 (-0.77 to 2.25) | 0.63 – 0.91 | -1.53 to 3.28 |
| Total cholesterol | -0.29 (-1.03 to 0.45) | -0.33 – -0.24 | -1.12 to 0.52 |
| HDL | 0.32 (-0.18 to 0.83) | 0.27 – 0.35 | -0.28 to 0.96 |
| LDL | -0.42 (-1.00 to 0.16) | -0.46 – -0.35 | -1.10 to 0.23 |
| Glucose | -0.18 (-1.91 to 1.55) | -0.31 – -0.03 | -2.10 to 1.74 |
| Triglycerides | -0.38 (-1.22 to 0.47) | -0.42 – -0.31 | -1.30 to 0.52 |
| Systolic blood pressure | -0.41 (-1.83 to 1.01) | -0.49 – -0.35 | -1.93 to 1.10 |
| Diastolic blood pressure | -0.43 (-1.34 to 0.48) | -0.48 – -0.38 | -1.42 to 0.54 |

VO_2max_ Maximum oxygen uptake; P_max_ peak power of the cycle ergometer test; HDL High-density lipoprotein cholesterol; LDL Low-density lipoprotein cholesterol.

**Supplemental table 26.** The certainty of evidence according to GRADE.

| **No. of studies** | **Risk of bias** | | **Inconsistency** | **Indirectness** | **Imprecision** | **Publication bias**  **(Egger’s test)** | **No. of subjects** | | **Effect size (95% CI)** | **Certainty** | |
| --- | --- | --- | --- | --- | --- | --- | --- | --- | --- | --- | --- |
| **Maximum oxygen uptake (ml/kg/min)** | | | | | | | | | | | |
| 37 | | Unclear* | Not serious (moderate heterogeneity) | Not serious | Not serious  (upgrade due to ES) | Not serious  (p = 0.31) | LIT  562 | C  479 | 0.94   (0.74 to 1.13) | | ⨁⨁⨁⨁  High |
| **Maximum oxygen uptake (l/min)** | | | | | | | | | | | |
| **7** | | Unclear* | Not serious (moderate heterogeneity) | Not serious | Not serious  (upgrade due to ES) | Not serious  (p = 0.93) | 71 | 67 | 0.84  (0.36 to 1.33) | | ⨁⨁⨁⨁  High |
| **P_max_** | |  |  |  |  |  |  |  |  | |  |
| 9 | | Unclear* | Not serious | Not serious | Not serious  (upgrade due to ES) | Not serious  (p = 0.95) | 110 | 96 | 1.09  (0.86 to 1.31) | | ⨁⨁⨁⨁  High |
| **First ventilatory threshold** | | | | | | | | | | | |
|  | |  |  |  |  |  |  |  |  | |  |
| 5 | | Unclear* | Not serious (moderate heterogeneity) | Not serious | Not serious | Not serious  (p = 0.42) | 62 | 62 | 0.74  (0.26 to 1.22) | | ⨁⨁⨁  Moderate |
| **Total cholesterol** | | | | | | | | | | | |
| 20 | | Unclear* | Not serious (moderate heterogeneity) | Not serious | Not serious | Not serious  (p = 0.30) | 322 | 282 | -0.29  (-0.50 to -0.09) | | ⨁⨁⨁⨁  Moderate |
| **High-density lipoprotein** | | | | | | | | | | | |
| 21 | | Unclear* | Not serious | Not serious | Not serious | Some  (p = 0.01) | 323 | 282 | 0.32  (0.16 to 0.48) | | ⨁⨁  Low |
| **Low-density lipoprotein** | | | | | | | | | | | |
| 15 | | Unclear* | Not serious | Not serious | Not serious | Not serious  (p = 0.29) | 223 | 190 | -0.42  (-0.62 to -0.22) | | ⨁⨁⨁  Moderate |
| **Glucose** | | | | | | | | | | | |
| 13 | | Unclear* | Serious inconsistency (high heterogeneity) | Not serious | Not serious | Not serious  (p = 0.93) | 174 | 157 | -0.18  (-0.66 to 0.30) | | ⨁⨁  Low |
| **Triglycerides** | | | | | | | | | | | |
| 19 | | Unclear* | Not serious (moderate heterogeneity) | Not serious | Not serious | Some  (p = 0.04) | 298 | 250 | -0.38  (-0.60 to -0.16) | | ⨁⨁⨁  Low |
| **Systolic blood pressure** | | | | | | | | | | | |
| 17 | | Unclear* | Serious inconsistency (high heterogeneity) | Not serious | Not serious | Not serious  (p = 0.46) | 233 | 224 | -0.41  (-0.76 to -0.06) | | ⨁⨁  Low |
| **Diastolic blood pressure** | | | | | | | | | | | |
| 17 | | Unclear* | Not serious (moderate heterogeneity) | Not serious | Not serious | Not serious  (p = 0.26) | 233 | 224 | -0.43  (-0.68 to -0.18) | | ⨁⨁⨁  Moderate |

*Considered unclear due to cumulative volume of “some concerns” in most involved studies.

## Description of search strategy

Phrases that were used in the PubMed search: ("endurance training" or "low intensity endurance training" or "low intensity training" or "LIT" or "moderate intensity endurance training" or "moderate intensity training" or "MIT" or "low intensity exercise" or "moderate intensity exercise" or "long slow distance" or "high volume training" or "continuous endurance training" or "continuous training" or "aerobic exercise" or "aerobic training" or "physical training" or "physical exercise") and ("Aerobic fitness" or "cardiorespiratory fitness" or "endurance performance" or "VO_2max_" or "oxygen consumption" or "economy" or "oxygen cost" or "efficiency" or "ventilatory threshold" or "lactate threshold" or "aerobic threshold" or "physical fitness" or "cardiovascular" or "cardiometabolic" or "lipid*") AND ("random*" or "intervention" or "comparative") AND ((humans[Filter]) AND (English[Filter])). In the SPORTDiscus search, source types were “academic journals”, language was restricted to “English”, and the search phrases were the same as used in PubMed.

## Effect size analyses

Effect size (ES) was calculated as suggested in (52):

$$\text{ES}=\frac{D_{\text{LIT}}-D_{\text{Control}}}{SD_{\text{Pooled}}}\times c,$$

where $D=\text{Mean}_{post}-\text{Mean}_{pre}$, for both the LIT and control group, is the difference from pre to post for the considered variable, $c=1-\frac{3}{4(n_{\text{LIT}}+n_{\text{Control}})-1}$ is a correction factor associated with Hedge’s g, and

$$SD_{\text{Pooled}}=\sqrt{\frac{\left( n_{\text{LIT}}-1 \right)\times SD_{\text{preLIT}}^{2}+\left( n_{\text{Control}}-1 \right)\times SD_{\text{preControl}}^{2}}{n_{\text{LIT}}+n_{\text{Control}}-2}}$$

is the pooled standard deviation consisting of pre-state standard deviations of LIT ($SD_{\text{preLIT}}^{2}$) and control ($SD_{\text{preControl}}^{2}$) groups. The variance of ES was calculated as suggested in (52):

$$\text{Var}\left( \text{ES} \right)=2c^{2}\left( 1-r \right)\left( \frac{n_{\text{LIT}}+n_{\text{Control}}}{n_{\text{LIT}}\times n_{\text{Control}}} \right)\left( \frac{n_{\text{LIT}}+n_{\text{Control}}-2}{n_{\text{LIT}}+n_{\text{Control}}-4} \right)$$

$$\left( 1+\frac{\mathrm{ES}^{2}}{2\left( 1-r \right)\left( \frac{n_{\text{LIT}}+n_{\text{Control}}}{n_{\text{LIT}}{\times n}_{\text{Control}}} \right)} \right)-\mathrm{ES}^{2},$$

where correlation coefficient $r$ describes the similarity between the pre- and post- exercise training data across participants. It can be calculated from: $r=\frac{SD_{\text{pre}}^{2}+SD_{\text{post}}^{2}-SD_{\text{Change}}^{2}}{2\times SD_{\text{pre}}\times SD_{\text{post}}}$. As practically no studies included in the meta-analysis reported $SD_{\text{Change}}$ (or paired t-test value from which $SD_{\text{Change}}$ could also be calculated), coefficient $r$ was estimated for each outcome variable from the studies of the author’s laboratory and studies that were screened during the process (“almost eligible studies”). The number of included studies varied between 2 and 22, and they included 99–738 participants. The obtained $r$ value was rounded to the nearest 0.05 and reduced by 0.10 to be on the safe side. The final $r$-values for outcome variables were: 0.75 (VO_2max_, HDL, LDL, triglyceride), 0.70 (threshold, economy, total cholesterol, systolic and diastolic blood pressure), and 0.55 (glucose).

In the reference (53), it is said that:

“If it is not possible to compute a correlation from any of the included studies, one can either estimate it from historical data or use an approximate value. In the latter case, the most common value to use is 0.5.”

For example, in (54) $r=0.5$ is considered to be the lowest sensible pre-post test correlation, or otherwise “the pretest-posttest design is less efficient than a test based on just the final measurements.” In this meta-analysis, historical data was utilized to get a more rational estimation of r.

## Calculating the pooled absolute values

The tables 3–13 show pre-values (mean $\pm$ SD), mean within-group changes, and mean between-group changes for both control and LIT groups. For lipids and glucose, it is also tabulated whether the analysis was made from plasma (p), or serum (s), or it was not reported (nr).

All pooled mean values were calculated as weighted means from individual studies, the sample size being the weights. Similarly pooled pre-values SD were calculated as weighted SD from individual studies, the sample size being the weights. To calculate the pooled SD of change in control and LIT, first the following equation was applied in each individual study

$\text{SD}_{\text{change}}^{2}=\text{SD}_{pre}^{2}+\text{SD}_{post}^{2}-2*r*\text{SD}_{pre}*\text{SD}_{post}$,

where $r$ is the correlation between pre- and post-states, and they were estimated to be 0.75 (VO_2max_, HDL, LDL, triglyceride), 0.70 (threshold, economy, total cholesterol, systolic and diastolic blood pressure), and 0.55 (glucose) (see “Effect size analyses” section above). Pooled change in LIT and control group SD was then calculated as weighted SD from individual studies, the sample size being the weights.

The SD of change LIT vs. control was calculated as weighted SD of study-wise (sample sizes being the weights), so that it does not approximate population SD, but SD between the studies.

## Description of subgroup analyses

The subgroup analysis was made only if there were at least two studies in a subgroup. If a subgroup contained fewer than two groups, the corresponding row in the table was left empty. Prediction intervals were calculated only for subgroups that included at least three studies. In the table, the homogeneity value between subgroups was bolded when the p-value was at most 0.05.

There were only five studies that reported VT1 results, thus no subgroup analysis was conducted for this outcome.

The subgroups were formed in the following way:

**Sex**
*Groups:* Male/Female.
*Rationale:* Biological groups.

**Training volume (h/week)***Groups:* $\leq$2.5 h; 2.6–4; >4
*Rationale:* 2.5 to 5 h/week is the recommended amount of weekly moderate physical activity (55, 56). Since there were not enough studies (n = 3) with > 5 h weekly, 4 h was chosen as the second threshold.

**Duration of exercise (min)***Groups:* $\leq$30 min; 31–50 min; >50 min
*Rationale:* A 30-minute exercise session can be considered a lower threshold for duration in regular training (55). Double of this would be 60 min, but there were too few studies for this category (n = 7), so instead 50 min was chosen as an upper boundary.

**Training frequency (per week)***Groups:* $\leq$3; 3.1–4.9; $\geq$5 *Rationale:* Too few studies (n = 2) with $<$ 3, so $\leq$3 was chosen as a lower threshold.

**Exercise intensity (% VO_2max_)**
*Groups:* $<$50; 50–55; >55
*Rationale:* Literature suggests that 50% /VO_2max_ could be the lower threshold for adaptations (57). We had 60% /VO_2max_ as our upper boundary for low-intensity training, making 55% the midpoint between 50% and 60%.

**Banister’s TRIMP (arbitrary unit)**
*Groups:* $\leq$3; 3.1–4.4; $\geq$4.5
*Rationale***:** Physical activity guidelines recommends 2.5 h of weekly moderate-intensity physical activity (55,56). When estimating the recommended 2.5 weekly hours to be at intensity 50% HRR, this turns into 3 a.u. with Banister’s TRIMP. 4.5 was 50% more than this.
*Calculation:* Banister’s TRIMP = exercise duration $\times$ $\Delta$HR $\times$ $e^{b\Delta HR}$, where $\Delta HR$ = (HR_avg_-HR_rest_)/(HR_max_-HR_rest_), and $b$ = 1.92 for males and 1.67 for females (58). Here $\Delta$HR was estimated to be the prescribed intensity presented as % \VO_2max_ and divided by 100 (e.g., if intensity was 50% \VO_2max_, $\Delta$HR was estimated to be 0.50). The coefficient $b$ was estimated to be 1.795 (average of 1.67 and 1.92), if both males and females attended intervention.

**Duration of intervention***Groups:* $\leq$6; 7–11; $\geq$12
*Rationale:* Too few studies (n = 1) < 6 weeks, so $\leq$6 weeks was used. Upper threshold of 12 weeks is a double of 6.

**Age (y)***Groups:* $\leq$45; 45.1–65
*Rationale:* Above 65 y is considered elderly people (59), while middle age was considered to start at 45.

**Body mass index (kg/m^2^)***Groups:* $\leq$25; 25.1–34.9
*Rationale:* In literature, 25 is considered the upper threshold for normal weight, and 35 for class II obesity (55).

**VO_2max_ (ml/kg/min)***Groups:* $<$30; 30–40; $>$40
*Rationale:* It has been proposed that 40 ml/kg/min would be a threshold beyond which certain minimum exercise intensity for adaptations is needed (57). Furthermore, similar subgroups were used in a previous minimum intensity analyses (57).

**References**

1. Asikainen TM, Miilunpalo S, Oja P, Rinne M, Pasanen M, Uusi-Rasi K, et al. Randomised, controlled walking trials in postmenopausal women: the minimum dose to improve aerobic fitness? Br J Sports Med. 2002 Jun;36(3):189–94.

2. Baghaiee B, Botelho Teixeira AM, Tartibian B. Moderate aerobic exercise increases SOD-2 gene expression and decreases leptin and malondialdehyde in middle-aged men. Science & Sports. 2016 Jun;31(3):e55–63.

3. Berger NJA, Tolfrey K, Williams AG, Jones AM. Influence of Continuous and Interval Training on Oxygen Uptake On-Kinetics. Medicine & Science in Sports & Exercise. 2006 Mar;38(3):504–12.

4. Blond MB, Rosenkilde M, Gram AS, Tindborg M, Christensen AN, Quist JS, et al. How does 6 months of active bike commuting or leisure-time exercise affect insulin sensitivity, cardiorespiratory fitness and intra-abdominal fat? A randomised controlled trial in individuals with overweight and obesity. Br J Sports Med. 2019 Sep;53(18):1183–92.

5. Camargo MD, Stein R, Ribeiro JP, Schvartzman PR, Rizzatti MO, Schaan BD. Circuit weight training and cardiac morphology: a trial with magnetic resonance imaging. Br J Sports Med. 2008 Feb;42(2):141–5.

6. Eguchi Y, Ohta M, Inoue T, Honda T, Morita Y, Konno Y, et al. Effects of Transitory Stimulation Interval Exercise on Physical Function: A Randomized Controlled Pilot Study among Japanese Subjects. J UOEH. 2012;34(4):297–308.

7. Gonçalves R, Motta-Santos D, Szmuchrowski L, Couto B, M. Soares Y, De O. Damasceno V, et al. Combined training is not superior to strength and aerobic training to mitigate cardiovascular risk in adult healthy men. bs. 2022;39(3):727–34.

8. Gormley SE, Swain DP, High R, Spina RJ, Dowling EA, Kotipalli US, et al. Effect of Intensity of Aerobic Training on V˙O2max. Medicine & Science in Sports & Exercise. 2008 Jul;40(7):1336–43.

9. Gossard D, Haskell WL, Taylor CB, Mueller JK, Rogers F, Chandler M, et al. Effects of low- and high-intensity home-based exercise training on functional capacity in healthy middle-aged men. The American Journal of Cardiology. 1986 Feb;57(6):446–9.

10. Grieco CR, Swain DP, Colberg SR, Dowling EA, Baskette K, Zarrabi L, et al. Effect of Intensity of Aerobic Training on Insulin Sensitivity/Resistance in Recreationally Active Adults. Journal of Strength and Conditioning Research. 2013 Aug;27(8):2270–6.

11. Hiruntrakul A, Nanagara R, Emasithi A, Borer KT. Effect of once a week endurance exercise on fitness status in sedentary subjects. J Med Assoc Thai. 2010 Sep;93(9):1070–4.

12. Hu M, Kong Z, Sun S, Zou L, Shi Q, Chow BC, et al. Interval training causes the same exercise enjoyment as moderate-intensity training to improve cardiorespiratory fitness and body composition in young Chinese women with elevated BMI. Journal of Sports Sciences. 2021 Aug 3;39(15):1677–86.

13. Huang YC, Tsai HH, Fu TC, Hsu CC, Wang JS. High-Intensity Interval Training Improves Left Ventricular Contractile Function. Medicine & Science in Sports & Exercise. 2019 Jul;51(7):1420–8.

14. Huang YC, Hsu CC, Wang JS. High-Intensity Interval Training Improves Erythrocyte Osmotic Deformability. Medicine & Science in Sports & Exercise. 2019 Jul;51(7):1404–12.

15. Huttunen JK, Länsimies E, Voutilainen E, Ehnholm C, Hietanen E, Penttilä I, et al. Effect of moderate physical exercise on serum lipoproteins. A controlled clinical trial with special reference to serum high-density lipoproteins. Circulation. 1979 Dec;60(6):1220–9.

16. Kong Z, Zhang H, Nie J, Wen L, Shi Q, Ng SF, et al. Exercise Training Increases Serum Cardiac Troponin T Independent of Left Ventricular Mass. Int J Sports Med. 2022 Jun;43(06):505–11.

17. Lee MG, Park KS, Kim DU, Choi SM, Kim HJ. Effects of high-intensity exercise training on body composition, abdominal fat loss, and cardiorespiratory fitness in middle-aged Korean females. Appl Physiol Nutr Metab. 2012 Dec;37(6):1019–27.

18. LeMura LM, Von Duvillard SP, Andreacci J, Klebez JM, Chelland SA, Russo J. Lipid and lipoprotein profiles, cardiovascular fitness, body composition, and diet during and after resistance, aerobic and combination training in young women. European Journal of Applied Physiology. 2000 Aug 15;82(5–6):451–8.

19. Lucibello K, Parker J, Heisz J. Examining a training effect on the state anxiety response to an acute bout of exercise in low and high anxious individuals. Journal of Affective Disorders. 2019 Mar;247:29–35.

20. McDonald MP, Sanfilippo AJ, Savard GK. Baroreflex function and cardiac structure with moderate endurance training in normotensive men. Journal of Applied Physiology. 1993 May 1;74(5):2469–77.

21. McDowell S, Hughes R, Hughes R, Housh T, Johnson G. The Effect of Exercise Training on Salivary Immunoglobulin A and Cortisol Responses to Maximal Exercise. Int J Sports Med. 1992 Nov;13(08):577–80.

22. Meyer T, Auracher M, Heeg K, Urhausen A, Kindermann W. Effectiveness of Low-Intensity Endurance Training. Int J Sports Med. 2007 Jan;28(1):33–9.

23. Mousavi SM, Heidarianpour A, Tavassoli H. Aerobic Exercise Training Effects on Omentin-1, Insulin Resistance, and Lipid Profile Among Male Smokers. Research Quarterly for Exercise and Sport. 2023 Jul 3;94(3):880–5.

24. Murphy MH, Murtagh EM, Boreham CA, Hare LG, Nevill AM. The effect of a worksite based walking programme on cardiovascular risk in previously sedentary civil servants [NCT00284479]. BMC Public Health. 2006 Dec;6(1):136.

25. Poon ETC, Siu PMF, Wongpipit W, Gibala M, Wong SHS. Alternating high-intensity interval training and continuous training is efficacious in improving cardiometabolic health in obese middle-aged men. Journal of Exercise Science & Fitness. 2022 Jan;20(1):40–7.

26. Rogers MW, Probst MM, Gruber JJ, Berger R, Boone JB. Differential effects of exercise training intensity on blood pressure and cardiovascular responses to stress in borderline hypertensive humans: Journal of Hypertension. 1996 Nov;14(11):1369–75.

27. Roxburgh BH, Nolan PB, Weatherwax RM, Dalleck LC. Is moderate intensity exercise training combined with high intensity interval training more effective at improving cardiorespiratory fitness than moderate intensity exercise training alone? J Sports Sci Med. 2014 Sep;13(3):702–7.

28. Seifert T, Rasmussen P, Brassard P, Homann PH, Wissenberg M, Nordby P, et al. Cerebral oxygenation and metabolism during exercise following three months of endurance training in healthy overweight males. American Journal of Physiology-Regulatory, Integrative and Comparative Physiology. 2009 Sep;297(3):R867–76.

29. Shabani R, Izaddoust F. Effects of aerobic training, resistance training, or both on circulating irisin and myostatin in untrained women. Acta Gymnica. 2018 Jun 30;48(2):47–55.

30. Shaw BS, Shaw I. Compatibility of concurrent aerobic and resistance training on maximal aerobic capacity in sedentary males. Cardiovasc J Afr. 2009;20(2):104–6.

31. Shaw I, Shaw BS, Krasilshchikov O. Comparison of aerobic and combined aerobic and resistance training on low-density lipoprotein cholesterol concentrations in men. Cardiovasc J Afr. 2009;20(5):290–5.

32. Sittiwicheanwong R, Ariyapitipun T, Gulsatitporn S, Nopponpunth V, Abeywardena M, Dahlan W. Alterations of atherogenic low-density lipoproteins and serum fatty acids after 12 week moderate exercise training in sedentary Thai women. Asia Pac J Clin Nutr. 2007;16(4):602–8.

33. Sloan RP, Shapiro PA, McKinley PS, Bartels M, Shimbo D, Lauriola V, et al. Aerobic Exercise Training and Inducible Inflammation: Results of a Randomized Controlled Trial in Healthy, Young Adults. JAHA. 2018 Sep 4;7(17):e010201.

34. Soori R, Rezaeian N, Khosravi N, Ahmadizad S, Taleghani HM, Jourkesh M, et al. Effects of water-based endurance training, resistance training, and combined water and resistance training programs on visfatin and ICAM-1 levels in sedentary obese women. Science & Sports. 2017 Jun;32(3):144–51.

35. Stein RA, Michielli DW, Glantz MD, Sardy H, Cohen A, Goldberg N, et al. Effects of different exercise training intensities on lipoprotein cholesterol fractions in healthy middle-aged men. American Heart Journal. 1990 Feb;119(2):277–83.

36. Stein PK, Boutcher SH. The effect of participation in an exercise training program on cardiovascular reactivity in sedentary middle-aged males. International Journal of Psychophysiology. 1992 Dec;13(3):215–23.

37. Suter E, Marti B, Gutzwiller F. Jogging or walking—Comparison of health effects. Annals of Epidemiology. 1994 Sep;4(5):375–81.

38. Tan S, Wang J, Cao L, Guo Z, Wang Y. Positive effect of exercise training at maximal fat oxidation intensity on body composition and lipid metabolism in overweight middle‐aged women. Clin Physio Funct Imaging. 2016 May;36(3):225–30.

39. Thompson D, Markovitch D, Betts JA, Mazzatti D, Turner J, Tyrrell RM. Time course of changes in inflammatory markers during a 6-mo exercise intervention in sedentary middle-aged men: a randomized-controlled trial. Journal of Applied Physiology. 2010 Apr;108(4):769–79.

40. Tsai JC, Chang WY, Kao CC, Lu MS, Chen YJ, Chan P. Beneficial effect on blood pressure and lipid profile by programmed exercise training in Taiwanese patients with mild hypertension. Clinical and Experimental Hypertension. 2002 Jan;24(4):315–24.

41. Tsai JC, Liu JC, Kao CC, Tomlinson B, Kao PF, Chen JW, et al. Beneficial effects on blood pressure and lipid profile of programmed exercise training in subjects with white coat hypertension. American Journal of Hypertension. 2002 Jun 1;15(6):571–6.

42. Tseng ML, Ho CC, Chen SC, Huang YC, Lai CH, Liaw YP. A Simple Method for Increasing Levels of High-Density Lipoprotein Cholesterol: A Pilot Study of Combination Aerobic- and Resistance-Exercise Training. International Journal of Sport Nutrition and Exercise Metabolism. 2013 Jun;23(3):271–81.

43. Van Aggel-Leijssen D, Saris W, Homan M, Van Baak M. The effect of exercise training on β-adrenergic stimulation of fat metabolism in obese men. Int J Obes. 2001 Jan;25(1):16–23.

44. Wang J shyan, Jen CJ, Chen H ing. Effects of Exercise Training and Deconditioning on Platelet Function in Men. ATVB. 1995 Oct;15(10):1668–74.

45. Wang JS, Jen CJ, Chen HI. Effects of chronic exercise and deconditioning on platelet function in women. Journal of Applied Physiology. 1997 Dec 1;83(6):2080–5.

46. Wang JS, Li YS, Chen JC, Chen YW. Effects of Exercise Training and Deconditioning on Platelet Aggregation Induced by Alternating Shear Stress in Men. ATVB. 2005 Feb;25(2):454–60.

47. Wang JS, Chen WL, Weng TP. Hypoxic exercise training reduces senescent T-lymphocyte subsets in blood. Brain, Behavior, and Immunity. 2011 Feb;25(2):270–8.

48. Werner CM, Hecksteden A, Morsch A, Zundler J, Wegmann M, Kratzsch J, et al. Differential effects of endurance, interval, and resistance training on telomerase activity and telomere length in a randomized, controlled study. European Heart Journal. 2019 Jan 1;40(1):34–46.

49. Wu LH, Chang SC, Fu TC, Huang CH, Wang JS. High-intensity Interval Training Improves Mitochondrial Function and Suppresses Thrombin Generation in Platelets undergoing Hypoxic Stress. Sci Rep. 2017 Jun 23;7(1):4191.

50. Zhang H, Tong T, Qiu W, Wang, J, Nie, J, He, Y. Effect of high-intensity interval training protocol on abdominal fat reduction in overweight Chinese women: A randomized controlled trial. Kinesiology. 2015 Jan;47(1):57–66.

51. Meyer T, Auracher M, Heeg K, Urhausen A, Kindermann W. Does cumulating endurance training at the weekends impair training effectiveness? European Journal of Cardiovascular Prevention & Rehabilitation. 2006 Aug;13(4):578–84.

52. Morris SB. Estimating Effect Sizes From Pretest-Posttest-Control Group Designs. Organizational Research Methods. 2008 Apr;11(2):364–86.

53. Fu R, Vandermeer BW, Shamliyan TA, O’Neil ME, Yazdi F, Fox SH, et al. Handling Continuous Outcomes in Quantitative Synthesis. In: Methods Guide for Effectiveness and Comparative Effectiveness Reviews [Internet]. Rockville (MD): Agency for Healthcare Research and Quality (US); 2008 [cited 2025 Sep 9]. (AHRQ Methods for Effective Health Care). Available from: http://www.ncbi.nlm.nih.gov/books/NBK154408/

54. Follmann D, Elliott P, Suh I, Cutler J. Variance imputation for overviews of clinical trials with continuous response. Journal of Clinical Epidemiology. 1992 Jul;45(7):769–73.

55. American College of Sports Medicine. ACSM’s guidelines for exercise testing and prescription. Eleventh edition. 11th ed. Vol. 2022. Philadelphia: Wolter Kluwer;

56. Piercy KL, Troiano RP, Ballard RM, Carlson SA, Fulton JE, Galuska DA, et al. The physical activity guidelines for Americans. JAMA - Journal of the American Medical Association. 2018;320(19):2020–8.

57. Swain DP, Franklin BA. V̇O2 reserve and the minimal intensity for improving cardiorespiratory fitness. Medicine and Science in Sports and Exercise. 2002;34(1):152–7.

58. Morton RH, Fitz-Clarke JR, Banister EW. Modeling human performance in running. Journal of Applied Physiology. 1990;69(3):1171–7.

59. World Health Organization. World Report on Ageing and Health. World Health Organization; 2015.
